# How teacher behaviors and perceptions, air change rates, and portable air purifiers affect indoor air quality in naturally ventilated schools

**DOI:** 10.3389/fpubh.2024.1427116

**Published:** 2024-10-03

**Authors:** Tian Xia, Julia Raneses, Brixon Schmiesing, Raquel Garcia, Alison Walding, Richardo DeMajo, Amy Schulz, Stuart A. Batterman

**Affiliations:** ^1^Environmental Health Sciences, School of Public Health, University of Michigan, Ann Arbor, MI, United States; ^2^Michigan State University, East Lansing, MI, United States; ^3^Southwest Detroit Environmental Vision, Detroit, MI, United States; ^4^Health Behavior and Health Equity, School of Public Health, University of Michigan, Ann Arbor, MI, United States

**Keywords:** indoor air quality (IAQ), environmental justice (EJ), school, air purifier, particulate matter, teacher’s behavior

## Abstract

**Introduction:**

Many school buildings have inadequate ventilation, rudimentary if any air filtration, and aging and poorly maintained mechanical systems, all of which can lead to poor indoor air quality (IAQ). These issues are especially acute in environmental justice (EJ) communities where schools are located in polluted areas. This community-based participatory research examines how IAQ in naturally ventilated school buildings is affected by the use of air purifiers, air change rates, outdoor pollution levels, and teacher and staff behavior.

**Methods:**

IAQ assessments were performed at two schools in Detroit, Michigan, which included building walk-through inspections and continuous indoor and outdoor measurements of black carbon (BC), particulate matter (PM_10_ and PM_2.5_), carbon dioxide (CO_2_), air change rates (ACRs), temperature, humidity, and sound pressure levels. Air purifiers with usage monitors were then installed, and the IAQ assessments were repeated. Teachers were surveyed before and after purifier deployment.

**Results:**

At baseline, classrooms had low ACRs (0.58–1.38 h^−1^), moderate PM_2.5_ levels (2.8–8.9 μg/m^3^), highly variable PM_10_ concentrations (4.7–37.5 μg/m^3^), and elevated BC levels (0.3–0.7 μg/m^3^), reflecting emissions from local traffic, industry and other sources. The installation and use of purifiers reduced pollutant levels and the overall performance matched the predictions of a single compartment model. However, daily reductions varied widely among classrooms, reflecting differences in teacher behavior regarding the frequency of opening windows and the operation of purifiers, including differences in purifier fan speed settings and whether purifiers were used at night. Survey responses indicated that many teachers were aware of IAQ problems. The higher rates reported for health symptoms and dissatisfaction at one school may have lowered the teachers’ tolerance to noise and reduced purifier use.

**Discussion:**

The study helps explain the variation reported in prior studies using purifiers, and it reinforces the need to monitor IAQ and purifier use, use enhanced filtration and increase ventilation, and engage with teachers and school staff to support and maintain IAQ programs in schools.

## 1 Introduction

Indoor air quality (IAQ) in schools can affect the health and academic performance of children. Reports over the past two decades have noted that IAQ can affect respiratory symptoms, allergies, asthma exacerbation, cognitive function, attention span, and academic achievement (1–12). Indoor pollutants most commonly measured in school classrooms include fine particulate matter (PM_2.5_) and carbon dioxide (CO_2_). PM_2.5_ exposure has been associated with asthma (10, 13), rhinitis (13), reduced lung function (2), and respiratory inflammation (3), and PM_2.5_ can contain compounds including polycyclic aromatic hydrocarbons (PAHs) that increase cancer risk (3) and pathogens (pathogenic bioaerosols) that promote disease transmission, including influenza and SARS-CoV-2 (14, 15). CO_2_ serves as a measure of ventilation adequacy and IAQ. High CO_2_ levels have been associated with decrements in student decision-making performance (12), test scores (16), and attendance (17), although evidence for adverse cognitive effects is inconsistent at moderately elevated CO_2_ levels (e.g., <2000 ppm) (18). Guidelines and reference values for a number of indoor pollutants have been established by the World Health Organization (WHO) (19) and the U.S. Environmental Protection Agency (USEPA) (20), but indoor monitoring and enforcement are uncommon. Often, PM_2.5_ and CO_2_ levels in classrooms exceed reference values, especially in less developed regions such as central Europe (21, 22) and China (23). In the U.S., attainment with reference levels has been mixed, e.g., 16 city schools in mid-Atlantic cities mostly met WHO guidelines for PM_2.5_ (25 μg/m^3^ daily average, the interim target 4) (19, 24) and the ASHRAE guidance for CO_2_ (1,000 ppm) (25), while pollutant levels tended to be higher in schools in the Midwest (26, 27).

Ventilation and filtration are the major and often effective methods to manage IAQ. Mechanical and natural ventilation (including infiltration and flows through door and window openings) brings in fresh (outdoor) air with low CO_2_ and, ideally, low concentrations of PM_2.5_ and other pollutants to dilute and displace indoor pollutants. Improved ventilation has been associated with improved student performance (4, 28) and reduced illness absence (29). Unfortunately, ventilation rates in many schools are low (30, 31). The effectiveness of filters integrated in mechanical ventilation systems or used in free standing air purifiers has been evaluated using measurements (32–34) and computational modeling (35). For example, indoor-to-outdoor (I/O) ratios of PM_2.5_ levels, an indicator of indoor removal and filter effectiveness, were predicted to dramatically decrease using MERV14 filters, while MERV8 filters, still the prevalent filter rating used in schools and offices, had only modest removals (32). In contrast, a Dutch study showed only 30% reductions in PM_2.5_ levels using MERV14 filters (34). HEPA filters, the highest efficiency filter type widely available, were sparingly used in schools until the COVID-19 pandemic when they were widely deployed in standalone air purifiers to help limit the transmission of SARS-CoV-2 viral particles (36, 37). In schools near traffic and airports, HEPA purifiers have been shown to lower levels and I/O ratios of ultrafine particles (38), although the number of symptom-days due to asthma did not change in schools in the northeastern US (39). Overall, IAQ measurements and health outcomes found in school studies vary considerably among studies, schools, and the classrooms within a school, and HEPA and other filters generally have not achieved the expected reduction in PM_2.5_ levels. Such results can be caused by many factors, including faulty assumptions regarding pollutant sources, ventilation and air flows; lower than expected performance of filters; measurement errors including the determination of IAQ and filter performance; and unanticipated behaviors including teacher and staff actions to open windows and turn off filters (40).

Naturally ventilated school buildings face more IAQ challenges than mechanically ventilated buildings in which indoor air can be filtered multiple times per hour, windows are typically closed, and ideally, air inlets are positioned in favorable locations that are away from traffic and other sources of pollution. In naturally ventilated buildings, IAQ is influenced by the joint effect of outdoor air pollution levels, meteorology, building characteristics and location, and occupant behavior. In addition, filtration may be absent, and pollutant sources, behaviors and room characteristics can all differ between rooms (41). Naturally ventilated classrooms rely on infiltration/exfiltration and opening windows and sometimes doors to increase ventilation and flush out indoor pollutants, including occupant-generated CO_2_. However, this also allows the entry of outdoor pollutants into the building without any filtration. Air purifiers can remove PM_2.5_ from outdoor sources such as traffic and wildfires, as well as indoor generated particles such as exhaled pathogenic aerosols and dust (42). However, balancing natural ventilation with purifier usage can be challenging (43). Key concerns of thermal comfort, cost, energy usage, noise and access can discourage window opening and lower purifier usage leading to poor IAQ. In most (80%) classrooms, windows are operated by teachers, who have higher comfort temperatures than children (44, 45), which can lead to insufficient ventilation and thermal discomfort among students. Filter costs (including purchase, electricity for operation, and maintenance) can be significant, thus, schools may opt out or abandon filter programs (37). Noise from air purifiers can disturb students and teachers (46), e.g., half of the students and teachers felt “rather disturbed” or “very disturbed” by the purifiers at a German high school (47). For reasons of noise, comfort (avoiding drafts), and cost, purifiers may be turned off or set to a low (and inadequate) flow rate (31, 40, 48). Window opening is less likely in city schools compared to suburban and rural schools (49). Notably, IAQ issues may be compounded in environmental justice (EJ) communities where economically-disadvantaged and/or minority children are likely to attend schools located near high-traffic roads (50, 51), the school buildings are old and deteriorated, and the poverty level of the community limits the availability and use of purifiers (49).

The objective in this community-based participatory research project is to understand the combined effects of teacher and staff perceptions and behaviors, air change rate and building configuration, the use of air purifiers using HEPA filtration, and outdoor air pollution on air quality in classrooms of naturally ventilated school buildings. In addition, teacher awareness of IAQ is evaluated and recommendations to improve IAQ at the schools are provided.

## 2 Methods

### 2.1 Community priorities and study initiation

In 2013, community and academic partners created the Community Action to Promote Health Environment (CAPHE) partnership with the objectives of addressing air pollution and promoting health equity in Detroit, Michigan. An early result of the partnership was the creation of a public health action plan, which included recommendations to improve air quality in schools and child-serving institutions, recognizing the importance of the school environment for children. The partnership developed guidelines for the Schools Indoor Environments Project (ScIP, https://caphedetroit.sph.umich.edu/information-air-quality/schools-indoor-environment-project/), which included prioritizing candidate schools situated near heavily trafficked roads or industry that served disadvantaged populations. Schools were identified and recruited by our partners. To promote understanding of the program, factsheets and other outreach materials were developed for students, teachers and parents, including plain language fliers in English and Spanish that described the program, PM_2.5_ pollution, and school environmental quality. We discussed the program with school administrators, including the benefits of air purifiers, which would be provided without cost to the school. In schools in which administrators expressed interest in participating in the ScIP, administrators were asked to introduce the program to the teachers during a regular staff meeting, sign a data sharing agreement, and provide a list of classrooms, teachers and emails, floor plans, hours of operation, and the best times and days to visit. The school administration was asked to encourage participation and compliance with study protocols. After completion of the study, we provided a report to administrators that summarized findings and recommendations, and followed up to answer any questions or concerns. The CAPHE Steering Committee, which includes 17 member organizations, provided input and direction throughout the study, and helped maintain ongoing engagement with the school and community regarding study progress and results.

This paper reports on two naturally ventilated schools in the larger ScIP study (which also included mechanically ventilated buildings). The selected schools are located in Detroit, Michigan, USA in densely populated areas that contain a wide range of industries and commercial facilities (e.g., refinery, coal-and gas-fired power plants, steel mills, a coking plant, gypsum and cement production facilities, car and truck assembly plants, sewage treatment facility, intermodal and logistics hubs). Additional air pollution sources include on-road traffic, including ~9,000 heavy-duty trucks that cross the Detroit US/Canada border daily, extensive traffic on surface streets, and widespread construction activities.

### 2.2 School inspection and assessment

**Figure 1:**
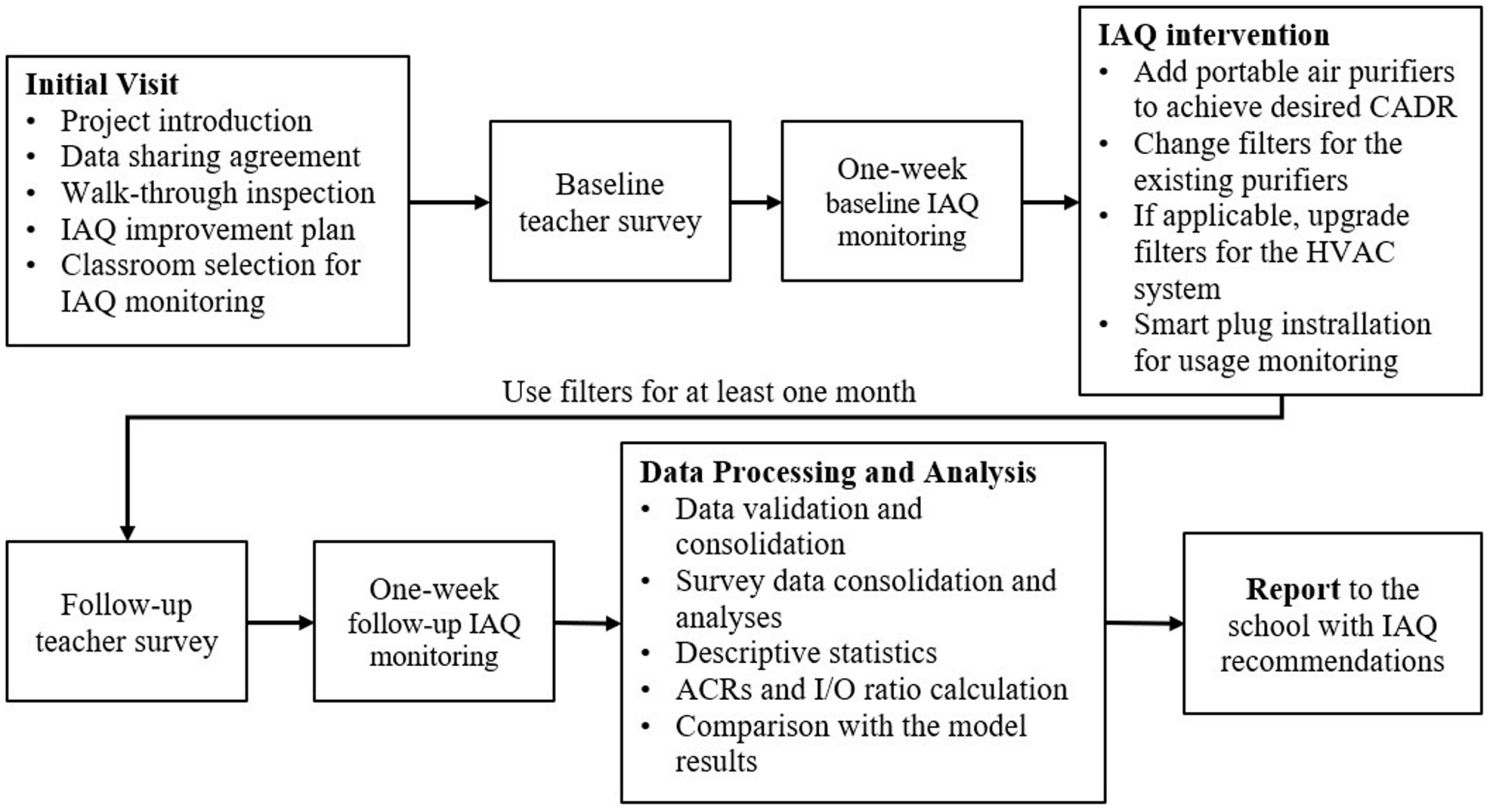
Overview of the approach showing monitoring, surveys, intervention and reporting.

An overview of the approach is shown in Figure 1. A walk-through visit of each school and classroom was conducted to document building and mechanical system features pertinent to IAQ, e.g., room dimensions, type and number of windows, heating/cooling systems, and number of students present. Three classrooms (designated R1, R2 and R3) were selected in each building along with one outdoor location to deploy monitoring equipment with the goal of continuous monitoring for at least five school days both before and after installation of air purifiers (described below). Sampler locations were selected to be representative, yet relatively unobtrusive. In most cases, samplers were placed near the center of an interior wall (generally at the rear of the classroom) away from windows and doors. To avoid disturbing occupants, sampling equipment was deployed and retrieved either after students had been dismissed or on non-school days. Typically, “baseline” monitoring was performed for one week, then purifiers were deployed, four to five weeks elapsed, and “follow-up” monitoring was performed for another week.

Seven types of instruments monitored indoor and outdoor environmental conditions. A custom-built case (to prevent tampering and damage to the instruments) contained an optical particle counter measuring PM_1_, PM_2.5_, PM_4_, PM_7_, PM_10_, and TSP (OPC; Aerocet 531S, Met One, Grants Pass, OR, United States); a 5-channel aethalometer measuring black carbon (BC; MA200, AethLabs, San Francisco, CA, United States); a sound pressure level (SPL) meter (SD-4023, Reed Instrument, Wilmington, NC, United States); a carbon dioxide (CO_2_) sensor (C7632A, Honeywell, Charlotte, NC, United States); and a combined temperature and relative humidity (T, RH) sensor (HOBO U10-003, Onset, Bourne, MA, United States). For these instruments, a small pump drew air through an inlet at the child’s breathing height (1 m) to instruments inside the case; the SPL microphone was at 1 m height. In addition, a second type of CO_2_ sensor (HOBO MX1102A, Onset, Bourne, MA, United States) with a larger CO_2_ range (up to 5,000 ppm) was used during follow-up visits at school A and for both visits at school B; and a second PM/black carbon instrument (ObservAir, DSTech, Berkeley, CA, United States) was used at school B. Outdoor air was simultaneously sampled using the same instrumentation (C7632A and MA200 for CO_2_ and BC measurements, respectively), although the outdoor data were limited as discussed below (Section 2.4). For school A, hourly PM_2.5_, PM_10_, BC, ambient temperature (T), relative humidity (RH), wind direction, and wind speed measurements at five nearby (within 4 km) stations were obtained and averaged to represent local conditions. SPL measurements were recorded at 1-s intervals, BC used 5-min integrated samples, PM used 1-min integrated samples taken every 5 min, and CO_2_, T, and RH were collected at 5 min intervals. The DST measurements were collected at 2-s intervals.

### 2.3 Teacher survey

Teachers and staff in the two schools were surveyed to obtain demographic information (age, sex, smoking status), health symptoms, comfort perceptions, and information related to IAQ and purifier use. The survey also included open-ended questions regarding health symptoms, comfort and indoor environment satisfaction. Each participant was provided with a link to the online survey and was requested to complete the survey during the baseline period (prior to the first round of IAQ monitoring). A second, nearly identical survey was administered several weeks after purifier installation and prior to follow-up monitoring. The school administrators encouraged survey participation, and reminder emails were sent to non-responders. All procedures followed the approved IRB protocol. While responses for baseline and follow-up periods were examined, the small sample size does not allow rigorous statistical testing, and in cases, results were pooled from two surveys.

### 2.4 Purifier deployment and monitoring

Air purifiers were installed in each classroom after the baseline monitoring. At school A, 8 Whispure air purifiers (Whispure, Honeywell, Charlotte, NC, United States) were installed, one in each of 8 classrooms on the wall opposite an existing air purifier (MA-40, Medify, Boca Raton, FL, United States), and three new Medify air purifiers were installed in two other classrooms as well as the special education room. The Whispure units have 4 speeds (low, medium, high, turbo) with corresponding CADRs of 221, 281, 320 and 404 ft^3^/min (375, 477, 544 and 686 m^3^/h, measured in a previous study) (52); the Medify units have 3-speeds (low, medium, high) and rated CADRs of 144, 181, and 239 ft^3^/min (245, 308, and 406 m^3^/h), respectively (53). Baseline measurements were collected in March 2022 and follow-up measurements in June 2022. At school B, 20 Whispure purifiers were installed, two on opposite sides of 10 regular classrooms. At this school, baseline measurements were taken in January 2023 and follow-up measurements in April 2023.

The number and CADR of the purifiers were selected to achieve an effective ACR of at least 3 h^−1^ (up to 6 h^−1^) in each classroom, following ASHRAE guidance (25) and using the combined flow of the purifiers and an assumed nominal natural ventilation rate of 0.5 h^−1^ (27). Considering the measured room volumes and the purifier CADR alone (i.e., excluding natural ventilation), effective ACRs in the classrooms ranged from 2.7 to 4.8 h^−1^ in school A and from 4.3 to 7.9 h^−1^ in school B.

Teachers were instructed to operate the purifiers at the highest setting they could tolerate without interfering noise issues during school hours, and they had the option to keep running the purifier overnight if they preferred. The power draw of each air purifier was logged every 10 min using a smart plug (Eve Energy, Munich, Germany).

### 2.5 Quality assurance and data analysis

Standardized methods and templates were used to clean and process the sensor and other data, facilitate quality assurance (QA), and implement calibrations. QA activities included the use of certified gas standards to calibrate the CO_2_ instruments, co-locating and calibration checks with new instruments to ensure measurement reproducibility, and checks with instrument diagnostics. For each data type, raw data were checked for allowable ranges, and averages were computed if at least 75% of the data for the period was valid. Ultimately, most data were reduced to 15-min averages. After calibration, the two types of CO_2_ sensors matched closely (e.g., slope = 0.90; intercept = 13 ppm; R^2^ = 0.96). The power consumption for each purifier fan speed was determined, and this calibration was applied to determine fan speed (~CADR). At school B, PM_2.5_ measurements using the DST instruments were 15–43% lower than the Aerocet measurements, and the Aerocet measurements are emphasized. The BC analysis at school B emphasizes the DST dataset as it is more complete.

Using the consolidated data for each classroom, trends were visualized and descriptive statistics were calculated. Air change rates (ACRs) were estimated for each classroom using the decay method (27). This entailed plotting the CO_2_ data to identify appropriate periods (trend with exponential-like decay for at least 30 min while occupied or 45 min while unoccupied, and a decrease of at least 200 ppm), fitting an exponential model (steady-state CO_2_ level, maximum CO_2_ level, decay rate) to the data using a constrained robust optimizer, and then averaging results across estimates for other periods in the day and study week. Results were considered valid if the model fit (as R^2^) exceeded 0.80 (R^2^ averaged 0.97) (Supplementary Figure S1 shows a screenshot and example CO_2_ plots used in the ACR calculation program). Typically, one CO_2_ decay curve was identified for the occupied period for each classroom per day, and a second for the unoccupied portion. Statistics for the school day when the buildings were normally occupied, typically from 8 a.m. through 3 p.m. were emphasized; statistics for unoccupied periods (evenings, weekends, and holidays) were also calculated. For both occupied and unoccupied periods, daily I/O ratios were calculated for each classroom using medians of the 15-min pollutant data, and the school-wide average across all sampling days and classrooms is reported.

A single compartment, steady-state model was used to estimate the expected reduction in classroom PM_2.5_ and BC levels from outdoor levels, accounting for particle size, classroom volume, purifier use and CADR, natural ventilation, and particle deposition. For evaluating the model, quasi-steady state periods were identified when indoor and outdoor pollutant concentrations remained relatively constant for at least one hour (which usually occurred between 10:00–12:00 or 14:00–16:00 while occupied and near midnight while unoccupied), providing an alternative metric to calculate I/O ratios and pollutant removals as compared to the use of median concentrations over the full occupied and unoccupied periods (noted above). The percentage reduction found indoors from outdoor levels was calculated as R = 100% × (1 – I/O). To allow comparison with the model, these calculations excluded a few periods when the I/O ratio exceeded 1 or when the ACR could not be estimated. The development and application of the IAQ model are detailed in the SI (Section 2).

Several sampling issues were encountered. At school A, electrical power to the outdoor sampler was unstable and much of the data was lost, thus synchronous measurements from five nearby monitors were acquired and analyzed to estimate outdoor levels. At school B, a suitable outdoor location with a power outlet was not available. Instead, the PM and BC monitors were placed in a school office and sampled outdoor air via a short piece (~ 1 m) of conductive tubing out the window; additionally, a DST instrument was mounted just outside the office window. One MA200 BC unit failed during the school A baseline monitoring, and a second failed during follow-up monitoring. Only two MA200 units were available during the baseline monitoring at school B, so the study relied on the DST BC instruments. At school A, electricity was unstable (particularly during the workday), power was lost on several occasions, and some of the smart plugs malfunctioned or were unplugged by teachers. However, data sufficient for analysis (>70% or ~ 10 school days) was recovered. The unstable power also caused some ambiguity in the power-fan speed relationship, causing some erroneous estimates of fan speed for the Whispure purifiers, e.g., ~8% of medium speed data was marked as low, 2% of high speed data was marked as medium, and 1% of turbo was marked as high. These errors are small and acceptable for the current study. At school B, similar smart plug problems occurred, and the smart plug in the basement classroom (R1) lost data for several weeks due to renovation activities. Again, most (>70% or ~ 13 school days) of the data were recovered.

## 3 Results and discussion

### 3.1 School inspection

School A is a single-story building constructed in the 1940s. It is located between major freeways with extensive vehicle traffic (over 100,000 vehicles daily, including over 10,000 commercial vehicles) (54), and it is near a busy arterial road and an intermodal facility with considerable diesel and train traffic. The school has a small, staff-only parking lot. Buses and private vehicles queue on local streets to pick up and drop off students. Classrooms are along a central corridor, and each has a floor area of 709 ft^2^ (65.8 m^2^) and volume of 8,050 ft^3^ (228 m^3^). A row of large single-pane windows covers most of the exterior wall; windows are openable in some rooms but blocked in others. An induction/radiator unit just below the windows spans this wall. These units contain an induction unit with fans blowing upward above a hot water radiator. They were designed to recirculate indoor and blend-in outdoor air from a wall-mounted air intake, however, air intakes were blocked off ~15 years earlier due to vermin (mice) infestation. The induction units do not have filters, although the radiator fins get caked with dust, which impedes airflow. A window air conditioner (AC) is installed in each classroom for cooling. On the interior wall, each classroom had two small ceiling-height vents to the central hallway. Nearly two years before the present study and during the COVID-19 pandemic, air purifiers had been deployed at the front of each classroom. Filters in these purifiers had not been replaced, some were not operating, and for the operating units, the lowest speed was typically used. The contents in the classrooms were typical for lower schools, including tables, chairs, small area rugs, books, computers, storage containers, posters, plants, markers, paint, glue, cleaning chemicals, art and science materials, and food (eating occurred in all classrooms).

School B is a two-story building with an occupied basement that was constructed in the 1920s. Like school A, this school is located on a residential block near two freeways and a moderately busy arterial. Private vehicles queue in the school parking lot and adjoining streets. The school includes regular and similarly sized special purpose classrooms for art, computer, and other activities. Classroom volume and area averaged 6,143 ft^3^ (174.0 m^3^) and 683 ft^2^ (63.4 m^2^), respectively (volumes ranged from 5,691–6,470 ft^3^, or 161.2–183.2 m^3^). All classrooms have openable windows, steam radiators served by a boiler in an outbuilding, and a window AC. A row of large double-pane windows covered most of the exterior walls, and most windows are openable. During the pandemic, a DIY air purifier had been installed in each classroom, which consisted of a box fan blowing upwards and placed in a cardboard jig above a 20 × 20 in MERV13 filter. At lower fan speeds, the flow rate was very low (<50 ft^3^/min; 85 m^3^/h); flows increased at high speed, but the system was very noisy. These units had been removed from most classrooms before the present study. Classroom contents were diverse and similar to those at school A. Some dirt and insects (ants) in less accessible places were observed.

In each school, classrooms held up to ~27 younger students (elementary and middle school) and 1 or 2 staff. Floors were vacuumed and mopped daily. Both carpets and/or hard surface (tile or wood) floors appeared clean with little accumulation of surface dust, and insects were found only occasionally in a few classrooms. In most cases, cleaning supplies and other chemicals were stored in cabinets, closets or a separate room, and few air fresheners or other indoor pollution sources were found. No significant evidence of water leaks, water intrusion, or flaking paint was found.

### 3.2 Teacher health and perceptions

Most teachers and staff in the schools completed both surveys, which are summarized in Table 1. At school A, respondents were mostly female (80%), all were full time (100%) and non-smokers (100%), although a few individuals had household members who smoked (12%). Over half of individuals reported health symptoms and discomfort experienced at work, most predominantly tiredness/fatigue, headache, difficulty concentrating, sneezing, back pain, and irritation of eyes, nose and throat, however, no respondents reported dizziness, wheezing, or breathing problems (with one exception, 3%). In most cases (74%), these symptoms went away overnight. While rates for tiredness/fatigue (58%) and headache (39%) were high, only a portion of individuals (37%) reported seeing a physician for these symptoms, and only one individual (4%) had taken time off work for pain or discomfort. Relatively few staff were dissatisfied with the thermal environment (21% in winter, 27% in summer), although overheating in winter and highly variable temperatures were common complaints (up to 36%). Regarding the indoor environment, few individuals were dissatisfied with the overall environmental quality (14%), but complaints due to general cleanliness, odors and noise were common. Several respondents noted that given the age of the building, they appreciated efforts to maintain and enhance environmental conditions. Odors were mostly related to food, perfume/cologne, and cleaning products; a few respondents also indicated body odor/stale air, chlorine and/or sewer gas. Dissatisfaction with noise was mainly due to fans, ventilation system, children, nearby classrooms or hallways, and air purifiers; noise from trucks and other vehicles was also mentioned. A minority of respondents (35%) indicated that poor air quality can interfere with the learning environment. Reported use of the air purifiers increased from 50% with the existing Medify purifiers at baseline to 75% with the Whispure purifiers in the follow-up period. As will be discussed below, monitoring of purifier use showed higher use rates, i.e., 64–94% for the three IAQ examined classrooms, and 68–90% across all classrooms.

**Table 1:** Summary of teacher survey collected at schools A and B.

Variable type	School A			School B		
Variable name	Baseline	Follow-up	Average	Baseline	Follow-up	Average
Demographics						
Number of Respondents (count)	17	16	17	12	12	12
Working at school for at least 3 years (%)	53	50	51	42	42	42
In classroom for at least 6 h per day (%)	94	88	91	83	83	83
Female (%)	78	81	80	67	58	63
Health symptoms experienced at school						
Respiratory (1) (%)	50	58	54	33	17	25
Cognitive (2) (%)	75	75	75	58	58	58
Ergonomic (3) (%)	38	50	44	25	25	25
Symptom goes away overnight (%)	77	71	74	75	100	88
Pain or discomfort caused time away from work (%)	8	0	4	0	0	0
Saw a doctor for school based symptoms (%)	46	29	37	38	29	33
Comfort						
Dissatisfied with thermal comfort in winter (%)	17	25	21	42	27	34
Too warm in winter (%)	35	31	33	75	50	63
Too cool in winter (%)	18	6	12	0	0	0
Too dry in winter (%)	18	6	12	33	25	29
Too humid in winter (%)	29	6	18	17	17	17
Temperature too variable in winter (%)	41	31	36	17	33	25
Too drafty in winter (%)	18	25	21	17	17	17
Dissatisfied with thermal comfort in summer (%)	33	20	27	42	36	39
Too warm in summer (%)	20	6	13	33	27	30
Too cool in summer (%)	33	13	23	17	18	17
Too dry in summer (%)	47	19	33	42	36	39
Too humid in summer (%)	0	0	0	8	9	9
Temperature too variable in summer (%)	33	44	39	50	36	43
Too drafty in summer (%)	33	13	23	50	45	48
Indoor environment						
Dissatisfied with overall environmental quality (%)	22	6	14	0	8	4
Dissatisfied with general cleanliness of classroom (%)	81	81	81	58	58	58
Dissatisfied with air quality (%)	33	13	23	8	8	8
Frequent odors (%)	47	31	39	17	8	13
Classroom has air freshener(s) (%)	19	25	22	17	17	17
Thermostat does not work (4) (%)	22	6	14	8	0	4
Portable heater(s) used (%)	28	31	30	8	0	4
Portable fan(s) used (%)	17	25	21	25	42	33
Air purifier usually turned on (%)	50	75	63	(5)	88	88
Air quality interferes with learning (%)	44	25	35	25	25	25
Dissatisfied with level of noise (%)	47	31	39	8	8	8
Dissatisfied with noise from air purifier (%)	36	17	26	0	0	0

Highlights shows responses that are greater than the other school, based on proportions tests at *p*-values of below 0.05 (orange) and 0.10 (beige). (1) Respiratory includes cough, shortness of breath, sneezing, irritation of eyes, nose throat, wheezing, sinus congestion, breathing problems. (2) Cognitive includes headache, tiredness/fatigue, difficulty concentration, blurred vision, dizziness. (3) Ergonomic includes hand pain and discomfort, back pain and discomfort, wrist pain and discomfort, neck pain and shoulder pain and discomfort. (4) Combines responses for winter and summer. (5) No air purifier at baseline for school B.

As at school A, most (63%) respondents at school B were female, all were full time and non-smokers (100%), and none had household members who smoked (0%). Symptoms and discomfort reported at work were similar to the other school, e.g., tiredness/fatigue, headache, sneezing, sinus congestion, and irritation of eyes, nose and throat. No respondents reported dizziness, wheezing, or breathing problems. Slightly over half of respondents (58%) reported some cognitive symptoms, e.g., tiredness/fatigue (46%) and headache (29%), but fewer than half of individuals reporting these symptoms had seen a physician for these symptoms, and none (0%) had taken time off work for pain or discomfort. A quarter (25%) reported some respiratory symptoms, most commonly sinus congestion (18%), sneezing (13%), and coughing (7%). Most teachers/staff (96%) did not express dissatisfaction with the environmental conditions in the school, and most staff considered their rooms “somewhat” clean (58%) and the remainder (42%) considered their rooms very clean. Satisfaction with the thermal environment was 61 and 66%, respectively, in warmer and cooler months. Drafts in summer were reported by nearly half (48%) of respondents. Staff were generally satisfied with air quality (92%). A quarter of the teachers/staff (25%) indicated that poor air quality can interfere with the learning environment. All but one respondent indicated that noise levels in their classroom were acceptable (92%), although some identified noise sources, which included echoes and reflection in their own classroom (21%), nearby classrooms and halls (21%), traffic (13%), and fans/ventilation systems (8%). None indicated a concern with the noise from the air purifiers. Finally, staff reported high use with the new purifiers (88% reported that the purifier was “always” on). As will be discussed below, use monitoring showed that filter use rates approached 100% (and at “high” speed) while occupied.

Differences between the two schools are highlighted in Table 1 using colors for statistically significant or near significant *p*-values. The two schools showed similar rates regarding teacher perceptions of comfort, but school A showed noticeably higher rates of respiratory, cognitive, and ergonomic symptoms, as well as greater dissatisfaction with the indoor environment, including cleanliness, air quality, odor and noise. While sample sizes were small and some results were not always consistent (e.g., ergonomic symptoms are not expected to be associated with environmental factors), the survey data suggests a higher level of dissatisfaction at school A. This might be attributed to poorer ventilation and air quality, but perceptions and survey responses can be influenced by many factors, and the schools differed in many ways. As examples, school A is more crowded (~40% more students), classrooms are more densely occupied (average in schools A and B: 2.4 and 3.2 m^2^/person; 8.1 and 8.7 m^3^/person), the racial/ethnic mix differs, and while no formal assessment was conducted, a higher level of work stress may be experienced at school A as suggested by the unanticipated turnover of the school administration. These perceptions might be linked to how teachers used the purifiers, e.g., stress and frustration with the workplace may lead to lower tolerance of noise and decreased compliance with instructions to utilize purifiers, as discussed next.

### 3.3 Air purifier usage

**Figure 2:**
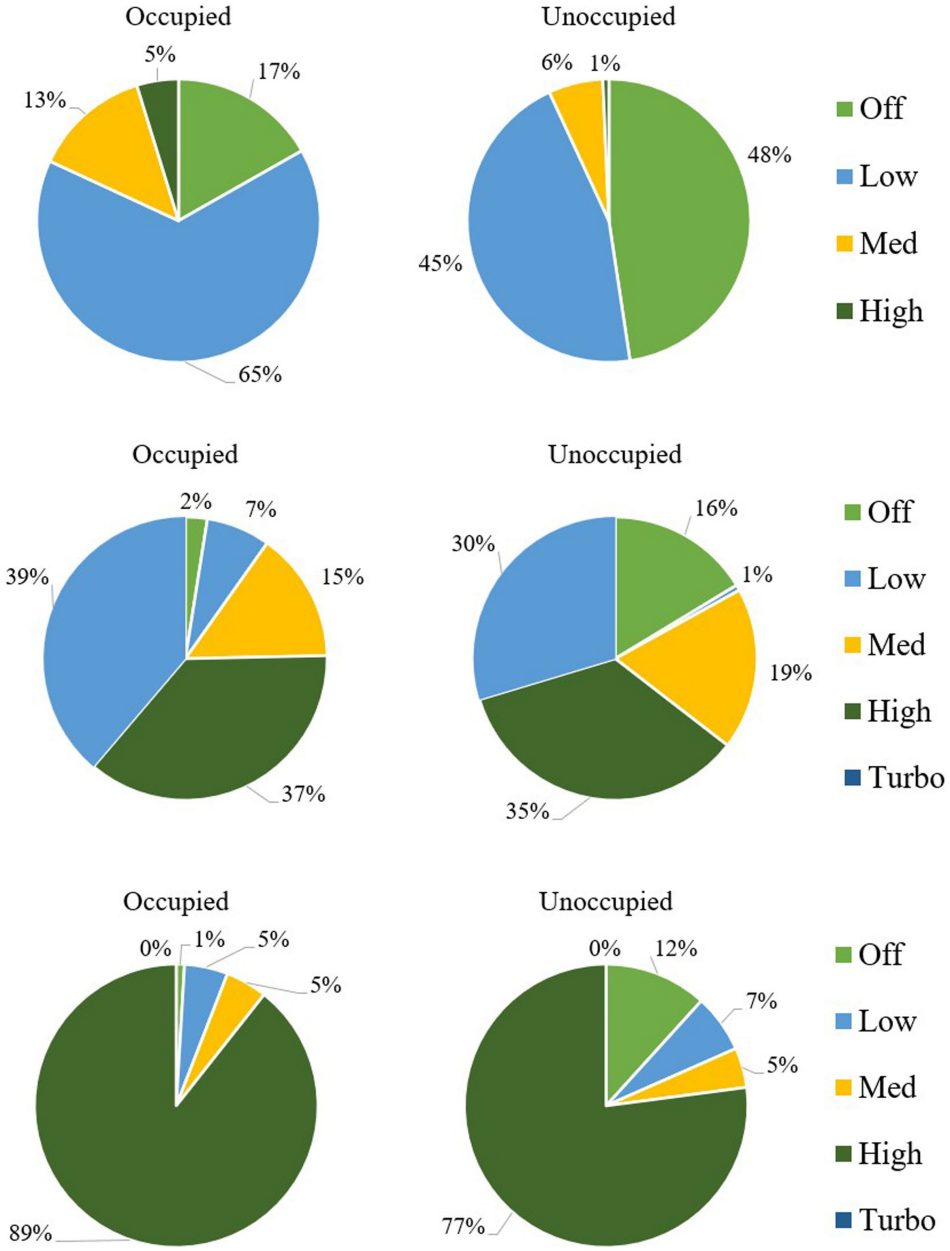
Purifier use and fan speeds at the two schools: **(A)** Medify purifiers at school A; **(B)** Whispure purifiers at school A; and **(C)** Whispure purifiers at school B. Shows occupied and unoccupied periods. Based on 71 occupied and 278 unoccupied hours of monitoring for school A, and 94 occupied and 543 unoccupied hours of monitoring for school B. School B had more unoccupied hours due to a one-week spring break.

The smart plug monitoring showed that after the intervention, purifier use at school A during the school day was generally high (averaging 90% of the time across the classrooms), but use depended on the classroom and filter type. At school A, the use rate dropped to 68% when the building was unoccupied (Figure 2). Almost all teachers turned off or adjusted the fan speed at least once per day, e.g., most teachers turned the purifiers off for some of the unoccupied periods, and several turned them off most evenings and then back on for the school day. Purifiers were often turned off during weekends. Several teachers kept purifiers off for several school days in a row. The two types of purifiers had distinct use patterns: the new Whispure units were used more frequently (average of 86%) and kept at “high” and “turbo” speeds (76 and 65% of occupied and unoccupied time, respectively), while use of the Medify units was lower (59%) and the most common speed was “low” (65% of occupied time and 45% of unoccupied time). At school B, purifiers were nearly always on (99% use) during school days. Teachers tended to operate both purifiers in their classroom in the same manner, and most used the “high” setting (88% of the time while the purifier was turned on), though two teachers used the “medium” rate due to noise. Most purifiers were left on during unoccupied periods (88%), although three teachers turned them off during week-long breaks and one teacher turned them off on weekends and some evenings.

The relatively low and inconsistent usage of purifiers at school A could result for many reasons, including noise disturbance (supported by survey results), electricity faults that shut off the purifiers (requiring teachers to manually turn them back on), teachers’ attention to other classroom issues, a lack of belief in the purifier’s effectiveness, and a lack of compliance with instructions for use that is possibly associated with communication issues. The more frequent use and higher speed setting for the Whispure purifiers might result from their location at the rear of the classroom, which caused less disturbance from drafts and noise, and made them less convenient to reach. The higher and more consistent purifier usage at school B might be due to teachers’ understanding and appreciation of the purifier’s effectiveness and benefits, and better communications between teachers and school leaders. While the factors affecting how teachers use purifiers cannot be definitively identified, purifier usage and thus teacher behavior clearly varied across classrooms, filter types and schools.

Earlier work examining purifier use in homes concluded that occupant responses on surveys regarding use and perceptions of filters frequently did not correspond to actual (monitored) purifier use, and that the use of purifiers must be considered an “active” intervention (40). These findings also may apply to school settings. Unknown and inconsistent use of purifiers may explain part of the variation seen in earlier school studies. Similar concerns apply to the many purifiers installed in schools during the COVID-19 pandemic. Understanding whether and how teachers are using purifiers is crucial in actions aimed at reducing exposure. Both energy cost (37) and noise (55) have been identified as impediments to purifier use in schools. A recent study also suggests the importance or nature of the underlying rationale, e.g., teachers concerned about disease transmission tended to use purifiers more consistently at the highest speeds than those concerned about wildfire smoke (56). More broadly, protection motivation and other behavioral health theories suggest that an individual’s perceptions of vulnerability, self-efficacy, response efficiency and other factors determine how individuals evaluate and respond or cope to threats such as poor air quality (57), although applications to IAQ are nascent. Overall, active interventions using purifiers would likely benefit from specific guidance and tools to encourage their use, including explaining their importance and impact, optimizing usage to achieve energy efficiency, and direct monitoring of use in research studies.

### 3.4 Baseline and follow-up air quality measurements

Air quality measurements at the schools are summarized in Table 2. CO_2_ levels in all classrooms were high, averaging 1845 ± 39 and 1,014 ± 63 ppm during occupied hours at schools A and B, respectively. Maximum levels (15-min average) exceeded 1,500 ppm in all classrooms, and two classrooms had peaks over 3,000 ppm. Baseline and follow-up levels were comparable. Thus, CO_2_ frequently and considerably exceeded the 1,000 ppm guideline (25).

**Table 2:** Summary of daily IAQ measurements and I/O ratios at the two schools for baseline and follow-up periods.

Parameter	Occupied		Unoccupied	
	Measurement	I/O Ratio^*^	Measurement	I/O Ratio^*^
**(A) School A**				
Baseline				
PM_2.5_ (μg/m^3^)	8.9 (3.7)	1.19 (0.64)	2.8 (1.2)	0.34 (0.08)
PM_10_ (μg/m^3^)	37.5 (7.2)	2.92 (0.72)	4.9 (2.2)	0.49 (0.30)
BC (μg/m^3^)	0.68 (0.23)	0.91 (0.23)	0.54 (0.20)	1.33 (0.32)
CO_2_ (ppm)	1845 (39)		647 (55)	
ACR (h^−1^)	0.58 (0.09)		0.58 (0.11)	
Follow-up				
PM_2.5_ (μg/m^3^)	2.9 (0.4)	0.28 (0.03)	1.4 (0.4)	0.17 (0.04)
PM_10_ (μg/m^3^)	20.0 (2.5)	1.04 (0.27)	3.5 (1.3)	0.20 (0.07)
BC (μg/m^3^)	0.33 (0.08)	0.37 (0.05)	0.44 (0.11)	0.74 (0.12)
CO_2_ (ppm)	1937 (196)		517 (52)	
ACR (h^−1^)	0.69 (0.12)		0.65 (0.10)	
**(B) School B**				
Baseline				
PM_2.5_ (μg/m^3^)	5.4 (1.3)	0.57 (0.07)	3.5 (1.0)	0.50 (0.23)
PM_10_ (μg/m^3^)	15.4 (1.6)	0.88 (0.24)	4.7 (1.1)	0.42 (0.10)
BC (μg/m^3^)	0.58 (0.09)	0.89 (0.13)	0.30 (0.08)	0.69 (0.11)
CO_2_ (ppm)	1,014 (63)		575 (16)	
ACR (h^−1^)	1.38 (0.21)		1.10 (0.30)	
Follow-up				
PM_2.5_ (μg/m^3^)	1.8 (0.9)	0.31 (0.09)	0.6 (0.2)	0.08 (0.03)
PM_10_ (μg/m^3^)	7.2 (1.0)	0.63 (0.17)	1.0 (0.3)	0.07 (0.02)
BC (μg/m^3^)	0.18 (0.05)	0.52 (0.17)	0.05 (0.03)	0.08 (0.03)
CO_2_ (ppm)	969 (83)		513 (28)	
ACR (h^−1^)	1.60 (0.29)		0.79 (0.16)	

Shows average and standard deviation (in parenthesis) of daily measurements in classrooms (*n* = 4 to 12). ^*^I/O ratios are dimensionless.

School-wide averages of indoor PM_2.5_, PM_10_ and BC concentrations and the I/O ratios for these pollutants were all substantially lower in the follow-up measurements with the air purifiers than the baseline values, although daily measurements in the classrooms varied considerably (discussed below in Table 2 and in the Supplementary Tables S1, S2). As examples, at baseline in school A, PM_2.5_ levels over the school day and across classrooms averaged 8.9 ± 3.7 μg/m^3^, slightly higher than outdoor concentrations (I/O ratio: 1.19 ± 0.64); BC averaged 0.68 ± 0.23 μg/m^3^, comparable to outdoor levels (I/O ratio: 0.91 ± 0.23); and PM_10_ averaged 37.5 ± 7.2 μg/m^3^, significantly higher than outdoor levels (I/O ratio: 2.92 ± 0.72). With the purifiers, I/O ratios for PM_2.5_, PM_10_ and BC during the school day dropped by 59 to 76%. The decrease in I/O ratios during unoccupied periods, 44 to 59%, was smaller, but purifier usage also decreased (90 to 68%). At school B, pollutant levels were lower (PM_2.5_: 5.4 ± 1.3 μg/m^3^, I/O ratio: 0.57 ± 0.07; PM_10_: 15.4 ± 1.6 μg/m^3^, I/O ratio: 0.88 ± 0.24; and BC: 0.58 ± 0.09 μg/m^3^, I/O ratio: 0.89 ± 0.13). With the purifiers, I/O ratios fell by only 28 to 46% with 100% purifier usage during the school day when some windows were opened; while I/O ratios during unoccupied periods fell by 83 to 88% with 88% purifier usage when most windows were closed (school B required teachers to close windows before leaving).

Using I/O ratios to evaluate *in situ* air purifier performance can help account for changing outdoor PM levels and PM characteristics that affect particle penetration efficiency through the building envelope and deposition rates. However, this indicator has several limitations. First, I/O ratios can vary considerably from day to day and between classrooms, particularly in naturally ventilated buildings, and rapidly changing indoor or outdoor levels (relative to the ACR) can increase uncertainty. In the Supplementary information, a second set of I/O ratios were determined for quasi-steady state periods (when pollutant levels were fairly constant), which somewhat reduced the variability but gave otherwise comparable results to the use of full day periods (Supplementary Figure S6). Second, given strong indoor sources and highly localized sources (and concentrations) of outdoor PM_10_ (58), I/O ratios generally do not reflect the performance of air purifiers or filtration for PM_10_. In classrooms, the coarse fraction of PM_10_ (PM_2.5–10_) arises mostly from indoor sources, e.g., soil and dust resuspended by cleaning and student movement, as highlighted by time trends of PM_10_ (including the difference between occupied and unoccupied periods, Table 2) and compositional differences (59). I/O ratios for PM_10_ may have some utility for schools located in very dusty or agricultural settings where outdoor PM_10_ levels may be high enough to dominate indoor levels, and I/O ratios of PM_10_ constituents can help identify specific emission sources (60). In contrast, PM_2.5_ and BC have few, if any, strong indoor sources in schools, and thus indoor levels reflect the penetration of outdoor pollutants into the space, even at the relatively low ACRs in the studied schools. For these pollutants, the change in I/O ratios associated with air purifiers from baseline to follow-up periods can be a good indicator of *in situ* purifier performance. Third and as discussed in the next section, I/O ratios for a given purifier will depend on the ACR. In comparing schools, the smaller change in I/O ratios at school B with purifiers likely reflects the higher ACRs at this school. This can apply building-wide, but in naturally ventilated buildings, window opening can alter pollutant levels in specific spaces, e.g., opened windows during follow-up measurements in school B allowed outdoor PM generated from vehicles queued in the school’s parking lot to enter into adjacent classroom R2 (Supplementary Figure S2). Other limitations can apply to I/O ratios of PM_2.5_ and BC: the ratios require local and representative estimates of ambient air quality and the use of data collected at distant or regional monitoring sites (and with different types of instruments) can introduce uncertainties, which may have affected results at school A.

### 3.5 ACRs and purifier removal rates

Table 2 summarizes ACRs at the two schools. At school A, the ACR averaged only 0.58 ± 0.09 h^−1^, and rates were fairly consistent (generally between 0.3–1 h^−1^); differences between occupied and unoccupied hours also were minor (window opening was uncommon at this school). At school B, ACRs averaged 1.38 ± 0.21 h^−1^ during the baseline period (range from 0.3 to 2.1 h^−1^), and increased slightly but not statistically in follow-up measurements when ACRs ranged from 0.3–2.5 h^−1^. The follow-up ACRs for the occupied period (0.7–2.5 h^−1^) were larger than those for unoccupied period (0.3–1.4 h^−1^), reflecting more frequent window opening during school hours in warm weather. Teachers were instructed by the school to close windows after school. Still, ACRs at both schools fell well below the recommended minimum of 3 h^−1^ (25).

ACRs in especially naturally ventilated buildings can be highly variable, affected by seasonal and short-term changes in outdoor temperature, wind speed and window openings. The main drivers of natural ventilation are the I/O temperature difference and wind speed (61). At both schools, baseline monitoring was conducted in winter or early spring, and follow-up measurements in late spring or early summer. Outdoor temperatures from baseline to follow-up increased at school A from an average of 3.5 to 22.5°C, but wind speed decreased from 14.4 to 11.7 km/h. At school B, temperatures increased from 1.7 to 10.9°C, and wind speed increased slightly from 12.4 to 13.4 km/h (62). As noted earlier, windows in some classrooms were partially opened during the warmer seasons, and after the school day, teachers closed windows in school B, though some windows in school A were left open overnight. Thus, despite the smaller I/O temperature differential in follow-up period, window opening (and wind speed at school B) were compensatory factors that acted to increase ACRs. Air purifiers may induce directional flows, promote mixing, and affect the temperature distribution in the classroom. While induced flows would not directly affect ACRs (63), changes in the indoor-outdoor temperature differential might affect the ACR, although only minimal effects are expected (64).

**Figure 3:**
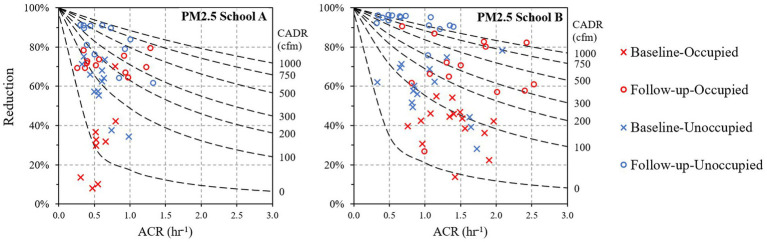
PM_2.5_ reductions at school **(A,B)** as a function of air change rate (ACR) in the six examined classrooms. Crosses and circles represent daily reduction estimates. Dashed lines represent the reduction as a function of ACR at different CADRs (0–1,000 ft^3^/min, or 0–1700 m^3^/h) from the steady-state model.

Predicted PM_2.5_ removal rates for the classrooms as a function of ACR and air purifier CADR are shown by the lines on Figure 3, which are based on a steady-state model assuming a single compartment (classroom), complete mixing, no indoor particle generation (PM_2.5_ and BC only from outdoor through ventilation and infiltration), and a single particle size (SI Section 2.1 describes the model development). The predicted removals approach 100% at very low ACRs as the entry or penetration of outdoor PM into the room is reduced, and as the CADR increases. Given that naturally ventilated classrooms typically have low ACRs, removals can be high with modestly sized filters, e.g., 80% removals can be achieved with a CADR from 200 to 250 ft^3^/min (340–425 m^3^/h) at a (low) ACR of 0.5 h^−1^, while 400 to 500 ft^3^/min (680–850 m^3^/h) is needed for an ACR of 1 h^−1^ (the CADR range reflects the variation in the classroom size).

Figure 3 also shows observed removal rates as points, estimated for each day and classroom using I/O ratios. The symbols’ color and shape separate baseline and intervention cases, as well as occupied and unoccupied periods. As noted earlier, daily removals of PM_2.5_ in each classroom varied considerably, depending on conditions in the classroom, e.g., window opening and ACR. At school A during the baseline period (Figure 3A), when Medify air purifiers were used in all three classrooms at low speed (~150 ft^3^/min (255 m^3^/h) CADR), PM_2.5_ removals across the three classrooms averaged 31 ± 10% (range: 8–70%; *n* = 9). Higher reductions were seen on several days in classrooms R2 and R3 when the Medify air purifiers were operated when the building was unoccupied (classroom R1 had the purifier turned off while unoccupied). In follow-up monitoring with the new purifiers (total CADR of 450–550 ft^3^/min (765–934 m^3^/h)), PM_2.5_ removals increased to an average of 72 ± 3% (range: 64–79%; *n* = 12) when the school was occupied, and 82 ± 4% (range: 62–91%; *n* = 12) when unoccupied. In room R1, the teacher turned off the purifiers during unoccupied hours, which lowered reductions (62–84%) compared to the other two classrooms (76–91%). At school B at baseline (Figure 3B), estimated PM_2.5_ reductions in the three classrooms while occupied averaged 40 ± 6% (range: 14–55%; *n* = 15). During the follow-up period, classrooms in school B had two Whispure purifiers, all operating at high speed (640 ft^3^/min (1,087 m^3^/h) CADR), which led to PM_2.5_ reductions that averaged 69 ± 9% (range: 27–90%, *n* = 14) during occupied periods, and higher and more consistent reductions of 92 ± 3% (range: 76–96%; *n* = 15) during unoccupied periods. Estimated BC reductions were generally lower than those seen for PM_2.5_ (Supplementary Figures S3A,B), e.g., for the occupied period, removals averaged 63 ± 5% (range: 43–72%; *n* = 8) at school A, and 54 ± 13% (range: 14–93%; *n* = 13) at school B. While BC removals tended to increase with higher CADR and with lower ACRs, uncertainties were large.

**Figure 4:**
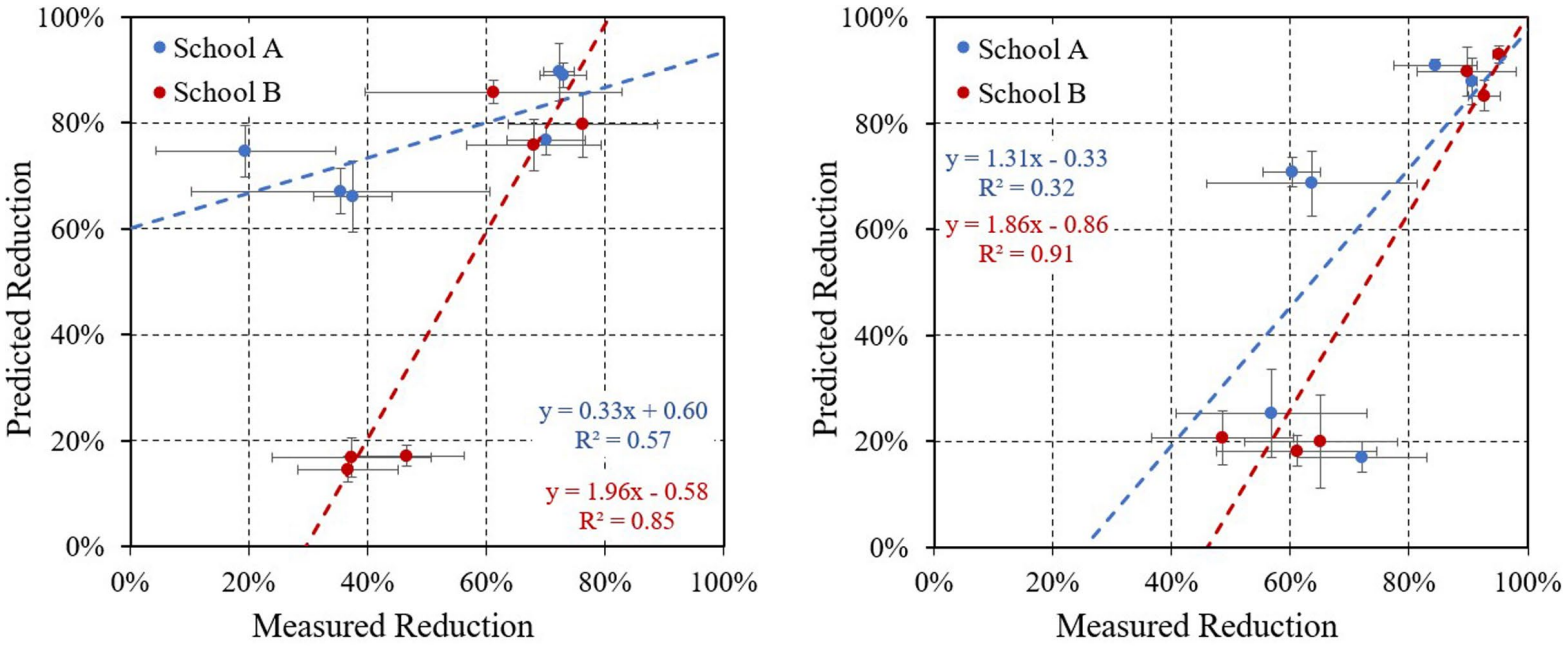
Predicted versus measured PM_2.5_ removal rates in six classrooms during **(A)** occupied and **(B)** unoccupied periods. Shows average and error bars for baseline and follow-up measurements at each classroom. The color of regression lines match the school symbols.

Predictions from the one-compartment model are contrasted with observed PM_2.5_ removal estimates in the scatter plot shown as Figure 4. At school A during baseline, PM_2.5_ removals were overpredicted during the school day and underpredicted when the building was vacant. At school B, estimates were highly correlated (R^2^: 0.85–0.91), possibly reflecting the more consistent use of the purifiers. Again, removal rates were underpredicted during the baseline when the building was either occupied or unoccupied. For BC, predicted reductions were generally overestimated at both schools during occupied hours, while predictions and measurements showed strong agreement at school B when either occupied or unoccupied and with all windows closed. The availability and uncertainty of the BC data limited the estimates at school A and increased variability at school B (additional details and the full BC analysis are presented in the Supplementary information; plots are shown in the Supplementary Figures S3C,D).

In practice, reductions estimated for PM_2.5_ and other pollutants will be affected by emission sources in classrooms, nonuniform mixing in the classroom, variation in the ACRs that is correlated with outdoor pollutant levels, and localized outdoor pollution and outdoor monitoring that is not representative of replacement air. These factors were not addressed by the single compartment steady-state model. The latter issue appears especially pertinent at school B, which had very wide hallways with high ceilings and few windows and doors, forming a second compartment with pollutant levels that would be attenuated from outdoor concentrations. Not accounting for the air exchange between classrooms and hallways would have the effect of increasing the measured reductions beyond predictions. In addition, results for classroom R3 in school B were especially variable during the follow-up period, probably due to this classroom’s proximity to a parking lot, briefly opened windows, and the entry of vehicle emissions. For BC, lower reductions were likely caused by data incompleteness, localized emissions (specifically from traffic) that elevated concentrations, and measurement uncertainty. At school A, the use of BC data from regional sites, rather than local measurements, further increased uncertainties. At school B, the BC sampler was installed on the east wall of the school near a street with limited traffic (due to construction closures), while the school’s parking lot and student pickup/drop-off area was to the northwest. With the predominant southwestern winds, vehicle emissions in the parking lot may not have been captured by the outdoor sampler, which is suggested by the larger reductions seen in the street-facing room (R1; 38–93% reductions) compared to reductions in the two parking-lot facing classrooms (14–72%). Despite the variability, the purifiers substantially reduced indoor pollutant levels, although their impact depended on day, classroom, school and purifier use and speed. The application of a single compartment model provided insight into the magnitude of expected reductions, but such models involve many simplifications and can sometimes yield large discrepancies between predicted and observed results.

### 3.6 Recommendations

While most individuals were not dissatisfied with the indoor environment, the IAQ measurements indicated several problems as well as opportunities to improve the indoor environment, specifically in terms of maintaining thermal comfort, reducing odors and PM levels, improving classroom ventilation, and reducing noise. As noted earlier, all sampled classrooms had low ACRs and high CO_2_ levels. One classroom in school A was noisy (SPL averaged 70 dB during occupied hours), and all classrooms in school B had low relative humidity (<30%) (20). The lack of awareness of these problems could lead to insufficient ventilation (e.g., not taking action to open windows) and not using the provided purifiers to reduce PM levels. However, many survey respondents acknowledged the importance of the indoor environment and utilized purifiers as requested.

Naturally ventilated school buildings have several options to improve classroom IAQ. Windows can be opened to achieve the suggested 3 h^−1^ air change rate and 1,000 ppm CO_2_ level cap (25), however, windows in classrooms adjacent to or affected by local pollutant sources (e.g., queued vehicles) should not be opened when the source is present (e.g., morning and afternoon drop-off and pickup periods). PM_2.5_, BC and other particulate pollutants brought indoors by increased ventilation, as well as internally generated particulate matter (including infectious aerosols), can be reduced with consistent operation of purifiers, even at low fan speeds (CADRs) that lessen draft and noise. Using purifiers during unoccupied hours (and with closed windows) can have a strong cleaning effect, but this wastes energy and filter life. More effective and “smarter” strategies might “preclean” the space using purifiers at a high CADR while keeping the windows shut for 30–60 min in the morning prior to occupancy (and during the drop-off period), utilize CO_2_ and PM monitoring to optimize filter operation, and message teachers to open or close windows. A few commercially available purifiers allow time programming (but only one “on/off” cycle per day), some utilize PM sensors to adjust CADR, and some record purifier use needed to ensure effective air cleaning. Different control strategies are needed for PM_10_, VOCs and other pollutants that have large indoor sources, e.g., PM_10_ can be controlled by damp mopping of floors, using HEPA-equipped vacuums, and minimizing dust generating sources and activities.

Future studies aimed at improving IAQ in classrooms might look at long-term trends in purifier use and performance, utilize larger samples, and examine performance in different climatic regimes. Given the importance of teacher behavior in naturally ventilated buildings, studies addressing the perceptions of indoor air risks, the benefits of IAQ literacy campaigns, and the adoption and use of protective strategies such as air purifiers are warranted, particularly in environmental justice areas where pollutant levels are high and building occupants are vulnerable to adverse impacts of air pollutants.

### 3.7 Study limitations

I/O ratios and measurements of purifier performance are affected by ACRs, meteorology, local pollution sources, measurement accuracy and representativeness, variable outdoor pollutant levels, teacher behavior in terms of filter use and opening windows, and other factors. Not all these factors could be measured directly or controlled. IAQ measurements were collected for one-week periods before and after the purifiers were installed, and day-to-day and seasonal variation may limit the long-term representativeness of measurements. Baseline and follow-up sampling periods were several months apart, and weather changes could alter ACRs, teacher behavior, and pollution levels. These variables were only partially controlled in the analysis using I/O ratios and estimated ACRs. The power instabilities and removal of smart plugs by teachers led to missing usage data. The single compartment model does not consider directional flows, indoor sources, and room-to-hallway exchange. Only two schools were examined, and the number of teachers surveyed was small. While the teacher survey investigated several factors that might affect behavior and attitudes, behavioral models were not used that might be helpful to promote protective behaviors, especially for relevant to the demographics and environmental conditions for schools in environmental justice areas. Despite these limitations, the study illustrates how teachers use portable purifiers and suggests the need to improve IAQ in naturally ventilated classrooms.

## 4 Conclusion

This community-based participatory research project was conducted at two naturally ventilated school buildings in an environmental justice area in Detroit, Michigan. At baseline, the studied classrooms had high CO_2_ levels, low air change rates, and PM_2.5_ and BC I/O ratios near 1 indicating little attenuation of outdoor pollutant levels, and surveys of teachers and staff indicated a lack of awareness of IAQ concerns. The operation of air purifiers effectively lowered particulate pollutants, however, measured removal rates varied widely among classrooms, and predicted removal rates were not achieved during the school day. A number of factors can affect removal rates, including potentially overlooked indoor sources, directional air flows, nonuniform mixing in the space, localized outdoor pollution, classroom-to-hallway air flows, unrepresentative pollutant and air change rate measurements, and teacher behavior in opening windows and using purifiers. The study suggests that IAQ in naturally ventilated classrooms can be improved using air purifiers, particularly if teachers are engaged, understand and support the IAQ program, and consistently use the purifiers. Purifier use appeared to increase with teachers who reported understanding the program and who were more satisfied with environmental conditions.

## Data Availability

The original contributions presented in the study are included in the article/Supplementary material, further inquiries can be directed to the corresponding author.

## Glossary

AC: Air conditioner
ACR: Air change rate
ASHRAE: The American Society of Heating, Refrigerating and Air-Conditioning Engineers
BC: Black carbon
CADR: Clean air delivery rate
CAPHE: The Community Action to Promote Health Environment
EJ: Environmental justice
HEPA filter: High efficiency particulate air filter
HVAC: Heating, ventilation, and air conditioning
I/O: Indoor/Outdoor
IAQ: Indoor air quality
MERV: Minimum efficiency reporting value
OPC: Optical particle counter
PAHs: Polycyclic aromatic hydrocarbons
PM: Particulate matter
QA: Quality assurance
RH: Relative humidity
ScIP: The Schools Indoor Environments Project
SI: Supplementary information
SPL: Sound pressure level
T: Temperature
USEPA: The U.S. Environmental Protection Agency
VOCs: Volatile organic compounds
WHO: World Health Organization
