# Supplementary material for: How teacher behaviors and perceptions, air change rates, and portable air purifiers affect indoor air quality in naturally ventilated schools
Source: Front Public Health. 2024 Oct 3;12:1427116. doi: 10.3389/fpubh.2024.1427116 (PMC11483887; doi:10.3389/fpubh.2024.1427116)
Supplement: Supplementary file 1 [file Data_Sheet_1.docx]

**How Teacher Behaviors and Perceptions, Air Change Rates, and Portable Air Purifiers Affect Indoor Air Quality in Naturally Ventilated Schools**

Supplemental Information

September 27, 2024

Tian Xia^1^, Julia Raneses^1^, Brixon Schmiesing^2^, Raquel Garcia^3^, Alison Walding^1^, Richardo DeMajo^4^, Amy Schulz^4^, Stuart A Batterman^1^

^1^Environmental Health Sciences, School of Public Health, University of Michigan, Ann Arbor, US

^2^Michigan State University

^3^Southwest Detroit Environmental Vision, Executive Director, Detroit, USA

^4^Health Behavior and Health Education, School of Public Health, University of Michigan, Ann Arbor, US

Contents

[1 Classroom indoor air quality (IAQ) variations at two schools 1](#_Toc178258023)

[2 IAQ measurement and single-zone model comparison 2](#_Toc178258024)

[2.1 Single-zone IAQ model 2](#_Toc178258025)

[2.2 Measured and predicted removal rates 3](#_Toc178258026)

[3 School environment description 4](#_Toc178258027)

[4 Instrumentation 4](#_Toc178258028)

[5 References 18](#_Toc178258029)

# Classroom indoor air quality (IAQ) variations at two schools

Indoor and outdoor air AQ quality measurements and I/O ratios for each classroom at the two schools are summarized in Table S1 and S2. These measures show considerable variation. Baseline PM_2.5_ at School A during occupied hours ranged 7.2 – 14.0 µg/m^3^, with 8.8 ± 5.8 µg/m^3^ outdoor concentration and 0.87 – 1.90 I/O ratios. With the air purifier, the classrooms showed similar PM_2.5_ concentrations, 3.2 – 3.4 µg/m^3^, with 9.8 ± 3.7 µg/m^3^ outdoors. The I/O ratio was consistent and ranged 0.27 from 0.31. PM_10_ baseline ranged 31.9 – 59.7 µg/m^3^, much higher than the 22.7 ± 19.8 µg/m^3^ outdoor concentration with 2.49 – 4.05 I/O ratios, indicating significant indoor sources such as fugitive dust. The follow-up measurements showed lower and more consistent concentrations among classrooms (19.2 – 27.4 µg/m^3^ and 0.81 – 1.21 I/O ratios) with similar outdoor pollution (23.9 ± 13.3 µg/m^3^). Baseline and follow-up BC measurements failed in classrooms R2 and R3 respectively. The available data showed similar levels between classrooms and a reduction of I/O ratio from 0.88 – 1.01 to 0.37 – 0.38 by the purifiers during occupied hours. PM levels were generally lower during unoccupied hours and indicated similar I/O ratio reductions by purifiers.

The variation in pollutant measurements at School A could have been caused by localized outdoor pollution sources, particularly near classroom windows, changes in classroom floor cleanliness and emissions, and measurement uncertainty. R2, the only road-facing classroom, had significantly higher PM_2.5_ concentrations and I/O ratios than the other two classrooms, suggesting localized traffic pollution. On some days (e.g., Tuesday) early morning or late afternoon peaks in PM_2.5_ levels were observed without a commensurate spike at the outdoor site. This might be due to local PM_2.5_ sources, possibly cleaning, smoking, or possibly cars and buses near the school dropping off and picking up students that did not affect the outdoor monitor sites. Peaks in the PM_10_ levels reflect indoor activities, e.g., classroom activities, student movement, etc., and generally did not correspond with outdoor levels. Peaks before and after the school day likely reflect cleaning activities (e.g., vacuuming). Indoor BC levels generally followed outdoor levels, showing the significance of outdoor emission sources. Some peaks in BC levels before and after the school day may be attributed to local traffic, including picking up and dropping students, using buses and other diesel-powered vehicles, however, BC also reflects ambient levels occurring throughout much of SW Detroit. The baseline I/O ratios for PM_2.5_ and BC were not statistically different from 1, suggesting that indoor and outdoor levels of these pollutants did not differ, a result of pollutant penetration into the building with little filtration and no internal sources. In contrast, the high I/O ratio for PM_10_ indicates the presence of emission sources in the classrooms, e.g., entrained dust. PM_2.5_ and PM_10_ I/O ratios fell during the unoccupied period, confirming the importance of school day activities. BC I/O ratios slightly increased during unoccupied period, possibly due to the rapid reduction of outdoor BC concentration at night.

At school B at baseline, PM concentrations were lower and classroom variations was smaller as compared to School A, with 5.1 – 5.7 µg/m^3^ PM_2.5_, 12.4 – 19.9 µg/m^3^ PM_10_, and 0.5 – 0.7 µg/m^3^ BC during occupied hours. Average outdoor PM_2.5_, PM_10_ and BC concentrations were 10.6, 21.4 and 0.74 µg/m^3^ respectively, and the corresponding I/O ratios were 0.52 – 0.60 for PM_2.5_, 0.64 – 1.03 for PM_10_, and 0.64 – 1.02 for BC. The follow-up measurements showed reduced PM levels but more classroom variations. PM_2.5_, PM_10_ and BC ranged 1.2 – 3.2 µg/m^3^, 4.4 – 12.5 µg/m^3^, and 0.1 – 0.3 µg/m^3^ respectively, with the average outdoor concentrations reduced to 5.7, 15.7 and 0.37 µg/m^3^ respectively. I/O ratios ranged 0.24 – 0.90 for PM_2.5_, 0.33 – 1.99 for PM_10_, and 0.33 – 0.63 for BC. Baseline unoccupied hours had similar PM_2.5_ and BC measurements as occupied hours, but considerably lower PM_10_ levels. The consistent use of purifiers during unoccupied hours noticeably reduced indoor PM concentrations and the corresponding I/O ratios in the follow-up monitoring.

Variations at School B were likely due to localized outdoor traffic emission events. Baseline outdoor PM_2.5_ levels on one Tuesday are somewhat atypical (Figure S2), possibly resulting from local sources. Spikes in PM_2.5_ levels around 8 AM appear in part attributable to traffic, including cars dropping off and picking up students at the school. Follow-up PM_2.5_ levels in classroom R2 (3.2 ± 3.7 µg/m^3^), while low, exceeded levels in the other two rooms (1.7 ± 0.8 µg/m^3^ and 1.2 ± 0.9 µg/m^3^), and trend plots (Figure S2) showed an atypical peak on Tuesday morning in classroom air. R2 is on the first floor and close to the parking lot. It is possible that the windows were left open in the morning and the classroom received significant level of PM_2.5_ from idling and queued vehicles dropping off the students. Outdoor measurements were collected southeast of the parking lot and may not have fully captured the idling emissions due to the predominant southwestern wind. This was further suggested by the elevated PM_2.5_ I/O ratio (0.90). PM_10_ and BC also showed elevated levels and I/O ratios on this day due to the suspected emission event. Because data for this day was unrepresentative, it was excluded in further I/O calculations and analyses. As in School A, peaks in the indoor PM_10_ levels at school B reflected indoor sources, e.g., student movement in classroom activities and cleaning, and BC levels generally followed outdoor levels, suggesting outdoor pollution penetration. Some peaks in BC levels before and after school may be attributed to local traffic emissions, while BC reflects changes in regional levels as noted above.

# IAQ measurement and single-zone model comparison

## Single-zone IAQ model

A single zone model predicted PM reduction due to filter use in the naturally ventilated classrooms. The model assumed a single particle size, a single zone (classroom), no indoor particle generation, and steady-state conditions. The governing equation is:

$\frac{\mathrm{VdC}}{\mathrm{dt}}=Q C_{0}-Q C-k {\eta CQ}_{c}- {\Sigma v}_{d,i} A_{i} C$ (1)

where V = classroom volume, C = PM concentration, t = time, Q = airflow rate due to natural ventilation, C_0_ = outdoor PM_2.5_ concentration, k = mixing factor representing the fraction of PM_2.5_ completely mixed with room air (usually 0.33 – 1.00) (1), η = purifier PM_2.5_ removal efficiency (99.97% for HEPA filters (2)), Q_c_ = air purifier CADR, v_d,i_ = deposition velocity to surface i, and A_i_ = deposition area. Measured classroom dimensions and volumes were used. Eq. 1 can be transformed to:

$\frac{\mathrm{dC}}{\mathrm{dt}}=r_{1} C_{0}-\left( r_{1}+k {\eta r}_{2}+D \right) C$ (2)

where r_1_ = classroom ACR, r_2_ = clean air delivery per hour (Q_c_/V) of the air purifiers, and D = overall deposition rate, defined by:

$D= \frac{v_{d,f}A_{f} + v_{d,w} A_{w} + v_{d,c} A_{c}}{V}$ (3)

where v_d_ = particle deposition velocity, A = surface area, and subscripts f, w and c refer to the classroom floor, four walls, and ceiling, respectively. Deposition velocities for a 2.5 μm diameter particle and a friction velocity of 0.3 m/s are used (3); this is the upper size limit for PM_2.5_ and thus represents a relatively high deposition scenario for fine fraction PM. Lai and Nazaroff (4) suggest a friction velocity of at least 0.1 m/s; we selected 0.3 m/s. Sensitivity analyses show that results are largely insensitive to still higher friction velocities (0.3 to 1 m/s; Fig. S4 vs. Fig. S5a).

At steady state, dC/dt = 0, and the fractional reduction in PM levels is a function of ACR and purifier usage:

PM_2.5_ Reduction = $1 - \frac{C}{C_{0}}=1- \frac{r_{1}}{r_{1} + k \eta r_{2} + D}$ (4)

We also modeled a wide range of CADRs (0 to 1000 ft^3^/min, or 0 – 1700 m^3^/hr) to show the variation in PM_2.5_ and BC removal rates. The mixing factor k was set to one, assuming a fully mixed condition. Figure S5 and S6 show a sensitivity analysis that compares k from 0.5 to 1.0. Results from eq. (4) were plotted with measured daily reductions (and error bars) for PM_2.5_ and BC concentrations for each monitored classroom and school.

## Measured and predicted removal rates

Predicted removal rates for PM_2.5_ and BC for each classroom for ACRs up to 3.0 hr^-1^ and CADRs from 0 to 1000 ft^3^/min (0 – 1700 m^3^/hr) are shown in Figure 2 and S3 along with the daily measurements for each classroom. The bottommost line in each panel reflects the no-purifier case (CADR = 0 ft^3^/min (0 m^3^/hr)) in which PM removal occurs by only particle deposition. Without filters and ACRs between 0.5 and 1.5 hr^-1^, typical of these naturally ventilated buildings, PM_2.5_ removal is between 15-30%, a result of deposition on building surfaces. Removals approach 100% at very low ACRs as penetration of outdoor PM into space is reduced, and increases with filters with CADR. However, increasing CADR from “low” to “turbo” fan speeds (CADR from 442 to 808 ft^3^/min (751 – 1373 m^3^/hr) using two purifiers) only modestly increases purifier effectiveness, e.g., removals increase from 81 to 88% at school B for 1 hr^-1^ ACR, and from 58 to 71% removal for a 3 hr^-1^ ACR (Figure S4). The predictions show that two Whispure purifiers should be able to reduce PM_2.5_ by 80% for ACRs below 1 hr^-1^, and by 60% for ACRs up to 3 hr^-1^ at school B, and the analysis suggests that in naturally ventilated buildings with low ACRs, only modest CADRs are needed to obtain high removals, e.g., 80%. This analysis assumes fully mixed (k = 1) and steady-state conditions. In practice, purifiers and fans in naturally ventilated spaces can produce directional flows and incomplete mixing that can lower removal rates. This may occur if the purifier recirculates local air (i.e., short-circuiting), ventilation and infiltration flows (containing PM) are preferentially distributed without being filtered, or if purifier exhaust (clean air) is preferentially exhausted, as can be shown using imaging techniques (5) and fluid dynamics models (6–9). In box models, a simple but incomplete approach to represent such effects uses a mixing factor that scales back ventilation or fan flows, however, spatial uniform concentrations are maintained (1). A sensitivity analysis showed that mixing factors k from 0.5 to 1 did not significantly change results (Figs. S5 and S6).

The data points in Figures 2a and 2b display the day-to-day variability in observed PM_2.5_ reduction rates. At school A prior to adding the purifiers, observed reductions are near the 0 ft^3^/min (0 m^3^/hr) CADR curve, as expected. With the purifiers, removals when the school was occupied fell between 100 and 500 ft^3^/min (170 and 850 m^3^/hr) CADR curves (actual CADR is 450 – 550 ft^3^/min (765 – 934 m^3^/hr) according to usage records), and removals when unoccupied were near or above the 500 ft^3^/min (850 m^3^/hr) CADR curve. At school B at baseline, most reductions fell near the 100 ft^3^/min (170 m^3^/hr) CADR curve; after the intervention, reductions when occupied were mostly between the 300 – 1000 ft^3^/min (510 – 1700 m^3^/hr) CADR curves, and when unoccupied, mostly above the 500 ft^3^/min (850 m^3^/hr, actual CADR is 640 ft^3^/min (1087 m^3^/hr)). Overall, this illustrates good agreement with model predictions for PM_2.5_. The agreement for BC was lower (Fig. S3a and S3b). At school A, use of regional BC levels increased uncertainties (I/O ratios ranged from 0.79 to 3.60 during baseline). Excluding I/O ratios above 1, baseline measurements fell near the 0 ft^3^/min (0 m^3^/hr) curve (n=4). Follow-up measurements when occupied mostly fell between 0 – 100 ft^3^/min (0 – 170 m^3^/hr) curves (7 of 8), and when unoccupied, mainly between 100 – 300 ft^3^/min (170 – 510 m^3^/hr) curves (4 of 7). School B had a more complete dataset, however, uncertainties increased at low BC levels (<0.3 μg/m^3^) which frequently led to I/O ratios exceeding 1. Here, most baseline reductions were near the 0 ft^3^/min (0 m^3^/hr) curve, and follow-up measurements, when the building was occupied, were between the 100 – 200 ft^3^/min (170 – 340 m^3^/hr)curves, and when unoccupied, above 750 ft^3^/min (1274 m^3^/hr).

For BC at school A, estimated baseline removals during occupied hours averaged 30 ± 9% (range: 5 – 45%, n = 6); in the follow-up measurements, reductions improved to 63 ± 5% (range: 43 – 72%, n = 8). School B had a more complete dataset. Here, BC levels were sometimes low (<0.3 μg/m^3^), which increased uncertainties and led to I/O ratios exceeding 1. During school hours, we estimated average reductions of 25 ± 8% (range: 4 – 69%, n = 11) at baseline, and 54 ± 13% (range: 14 – 93%, n = 13) in the follow-up measurements. Reductions tended to be higher during the unoccupied period.

# School environment description

School A is located on a residential street but is close to two large freeways and a moderately busy arterial and a huge freight terminal that has considerable diesel and train traffic. Buses and private vehicles queue on nearby streets to pick-up and drop-off children. The school has two parking lots for school staff. The single-story building was constructed in the 1940s. The building has a brick veneer, flat roof, and large single-pane windows covering most of the exterior walls.

School B is located in on an arterial road in a residential area. It is located near several busy roads including freeways, and a large freight terminal with extensive truck and train traffic is within approximately 1 km. Private vehicles queue in the school parking lot and adjoining streets to pick-up and drop-off children. The two connected buildings were built in the 1900s and have a brick veneer, pitched roof, and new large double-pane windows covering much of the exterior walls.

In addition to traffic pollution, air pollution sources that can affect both schools include the many industrial activities in southwest Detroit, extensive truck traffic, and fugitive dust from construction sites, materials handling and other sources.

# Instrumentation

Aerocet 531S, Met One

- Parameters: PM_1_, PM_2.5_, PM_4_, PM_7_, PM_10_, and TSP
- Unit: µg/m^3^
- Range: 0 – 1,000 µg/m^3^
- Resolution: 0.1 µg/m^3^
- Sensitivity: High = 0.3µm, Low = 0.5µm
- Measurement frequency: 1-min measurement for every 5 min

MA200, AethLabs

- Parameters: Carbonaceous particle under 880 nm (BC), 625 nm, 528 nm, 470 nm, and 375 nm wavelengths
- Unit: µg/m^3^
- Range: 0 – 1,000 µg/m^3^
- Resolution: 0.001 μg/m^3^
- Measurement frequency: 5 min

SD-4023, Reed Instrument

- Parameters: Sound pressure level (SPL)
- Unit: dB(A)
- Range: 30-130 dB
- Resolution: 0.1 dB
- Measurement frequency: 1 s

C7632A, Honeywell

- Parameters: CO_2_
- Unit: ppm
- Range: 0-2000 ppm
- Resolution: 1 ppm
- Measurement frequency: 5 min

HOBO U10-003, Onset

- Parameters: Temperature (T) and relative humidity (RH)
- Unit: T: °C; RH: %
- Range: T: -20° – 70°C; RH: 25% – 95%
- Resolution: T: 0.14°C at 25°C; RH: 0.07% @ 25°C and 30% RH
- Measurement frequency: 5 min

HOBO MX1102A, Onset

- Parameters: T, RH, CO_2_
- Unit: T: °C; RH: %; CO_2_: ppm
- Range: T: 0 – 50 °C; RH: 1 – 90%; CO_2_: 0 – 5,000 ppm
- Resolution: T: 0.024°C at 25°C; RH: 0.01%; CO_2_: 1 ppm
- Measurement frequency: 1 min

ObservAir, DSTech

- Parameters: BC (PM, CO, NO_2_, T and RH were measured but not used)
- Unit: µg/m^3^
- Range: 0 – 500 µg/m^3^
- Resolution: 0.001 µg/m^3^
- Measurement frequency: 2 s

Smart plug, Eve Energy

- Parameters: Power consumption
- Unit: Wh
- Measurement frequency: 10 min

Table S1. IAQ measurements and the corresponding I/O ratios for the three examined classrooms at School A

1. Baseline indoor measurements

1. Follow-up indoor measurements

1. Baseline outdoor air quality measured by the five nearby monitoring sites.

1. Follow-up outdoor air quality measured by the five nearby monitoring sites.

1. Baseline estimated I/O ratios.

1. Follow-up estimated I/O ratios.

Table S2. IAQ measurements and the corresponding I/O ratios for the three examined classrooms at School B

1. Baseline indoor measurements

1. Follow-up indoor measurements

1. Baseline outdoor air quality measured by our sampler.

1. Follow-up outdoor air quality measured by our sampler.

1. Baseline estimated I/O ratios.

1. Follow-up estimated I/O ratios.

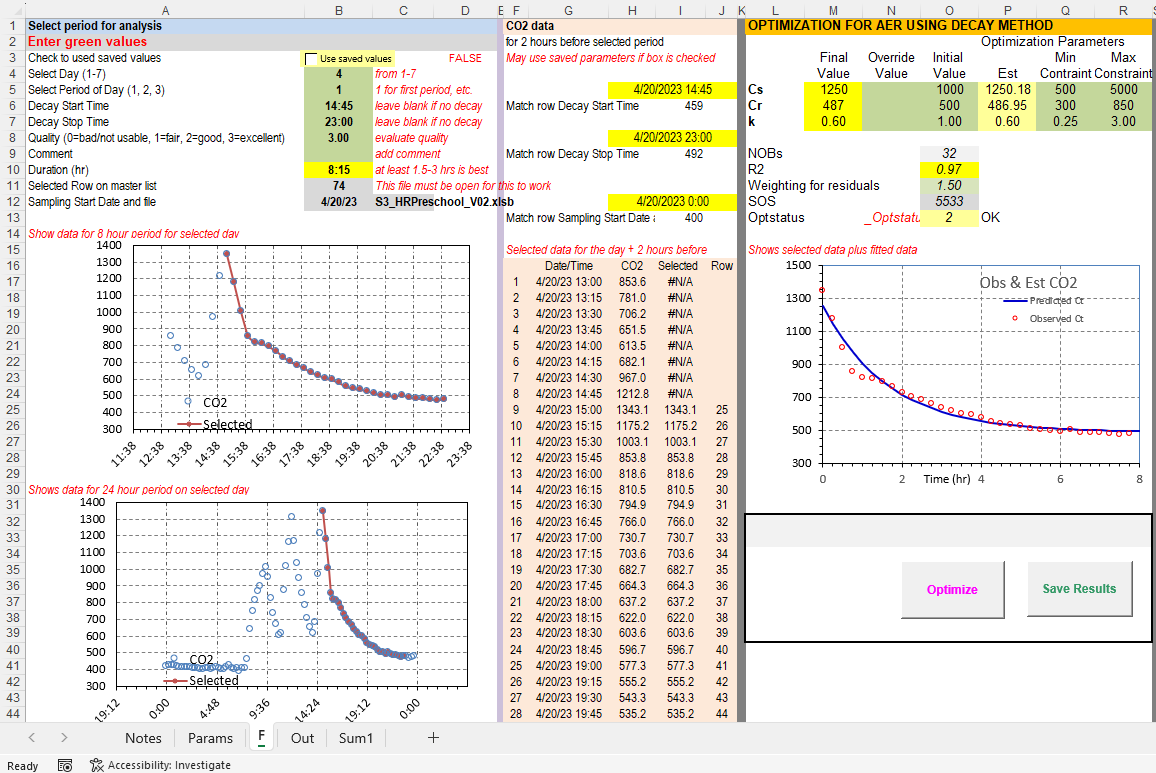


A)


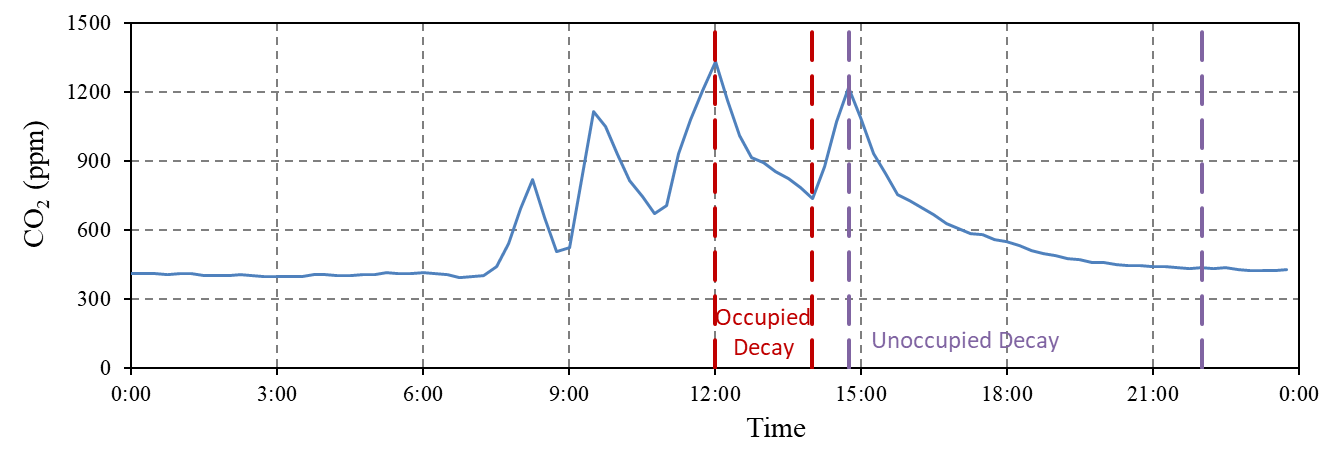


B)


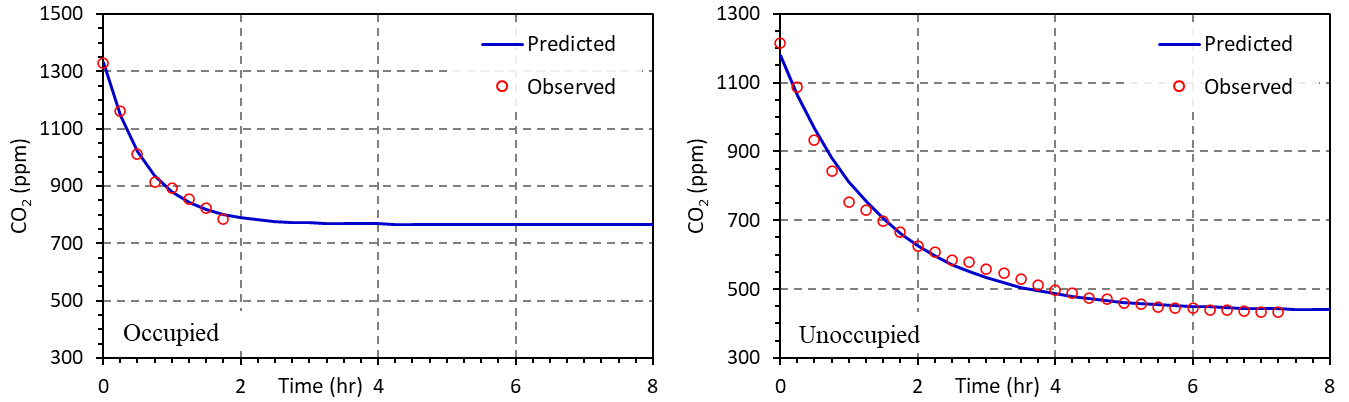


ACR = 0.68

r^2^ = 0.99

ACR = 1.60

r^2^ = 0.99

C)

Figure S1. A) Screenshot of the air change rate (ACR) calculation program in Microsoft Excel; B) Typical CO_2_ measurements during a school day at school B; C) fitted decay curves for occupied and unoccupied periods in that day.


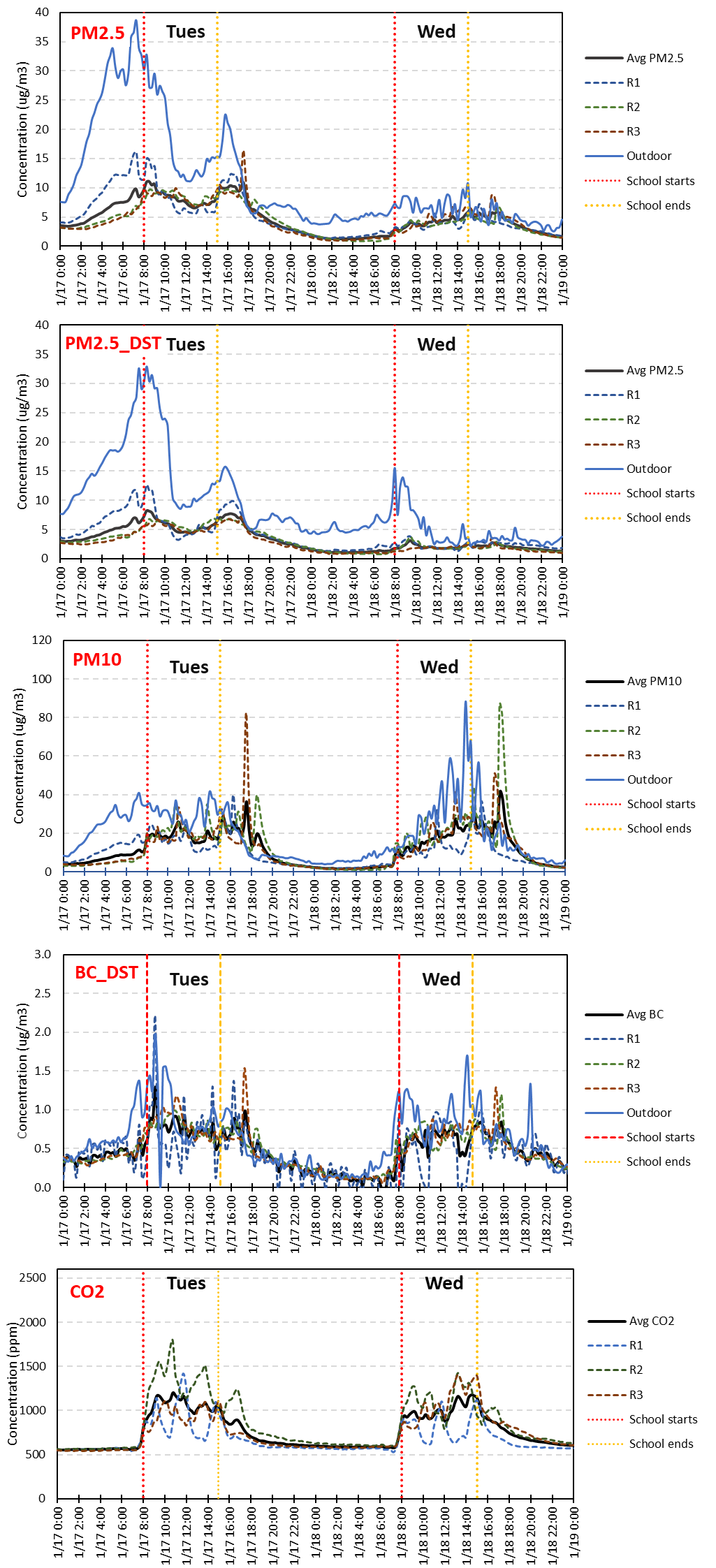

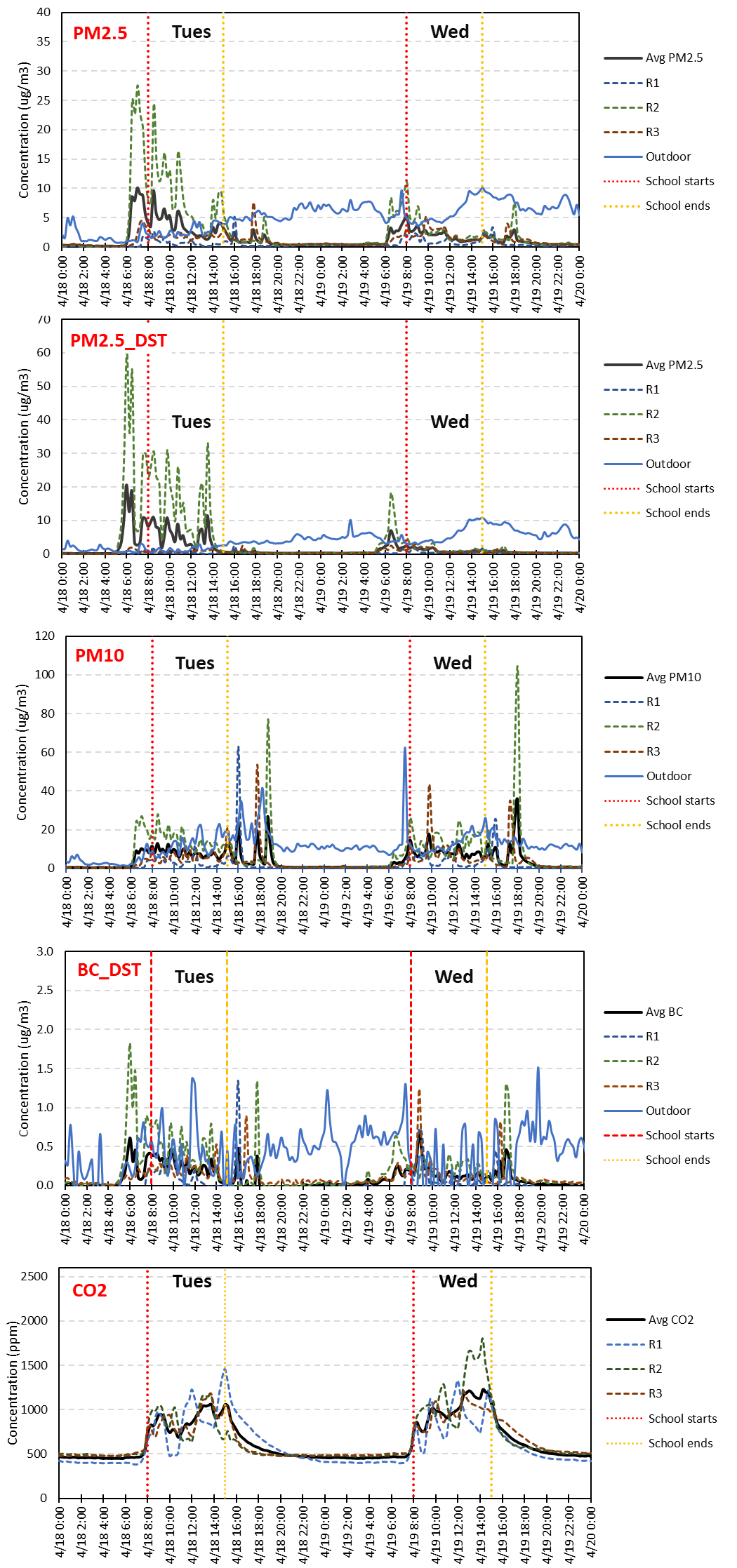


Figure S2. PM_2.5_, PM_10_, BC, and CO_2_ trends in the three classrooms and outdoors over two school days at School B. Left: pre-intervention; Right: post-intervention. Shows trends for each classroom and for outdoors. School day start and end shown by dashed vertical red and orange lines, respectively. Solid black line is the average concentration across classrooms; gaps indicate missing data, overrange or other instrument errors. Grade 2 was significantly polluted on Tuesday post-intervention, which could be due to emissions from idling cars outdoors. The outdoor sampler was installed on the other side of the building and thus did not capture this local event.


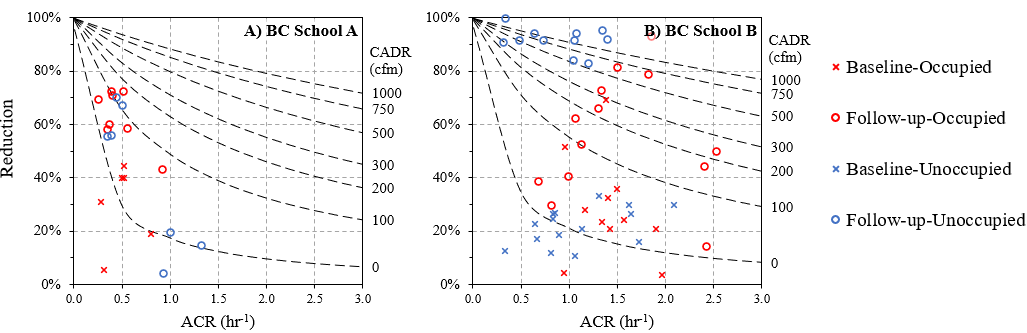


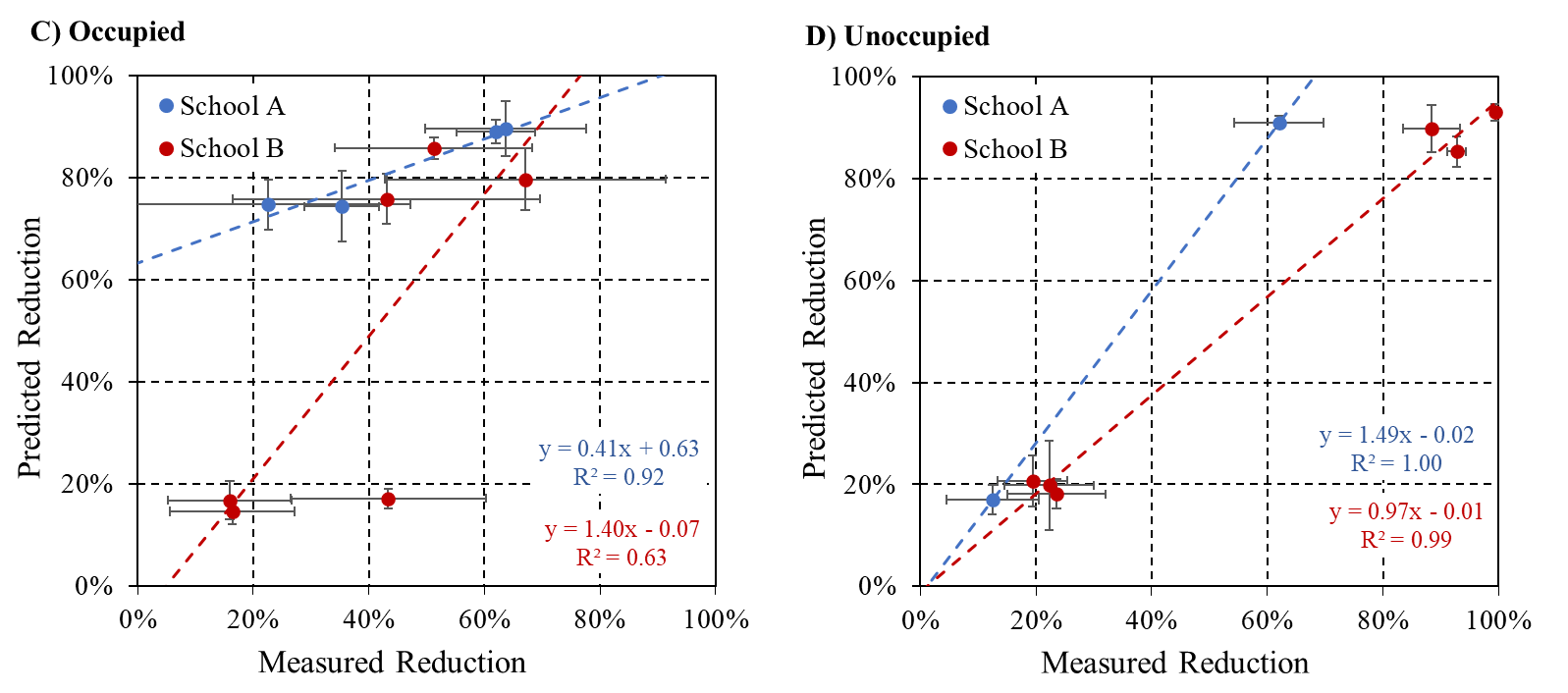


Figure S3. BC reductions at school A (a) and B (b) as a function of air change rate (ACR) in the six examined classrooms. Crosses and circles represent daily reduction estimates. Dashed lines represent predicted reduction as a function of ACR at different CADR (0 – 1000 cfm). Predicted versus measured BC removal rates in six classrooms during c) occupied and d) unoccupied periods were plotted as well. Shows average and error bars for baseline and follow-up measurements at each classroom. The color of regression lines matches school symbol.


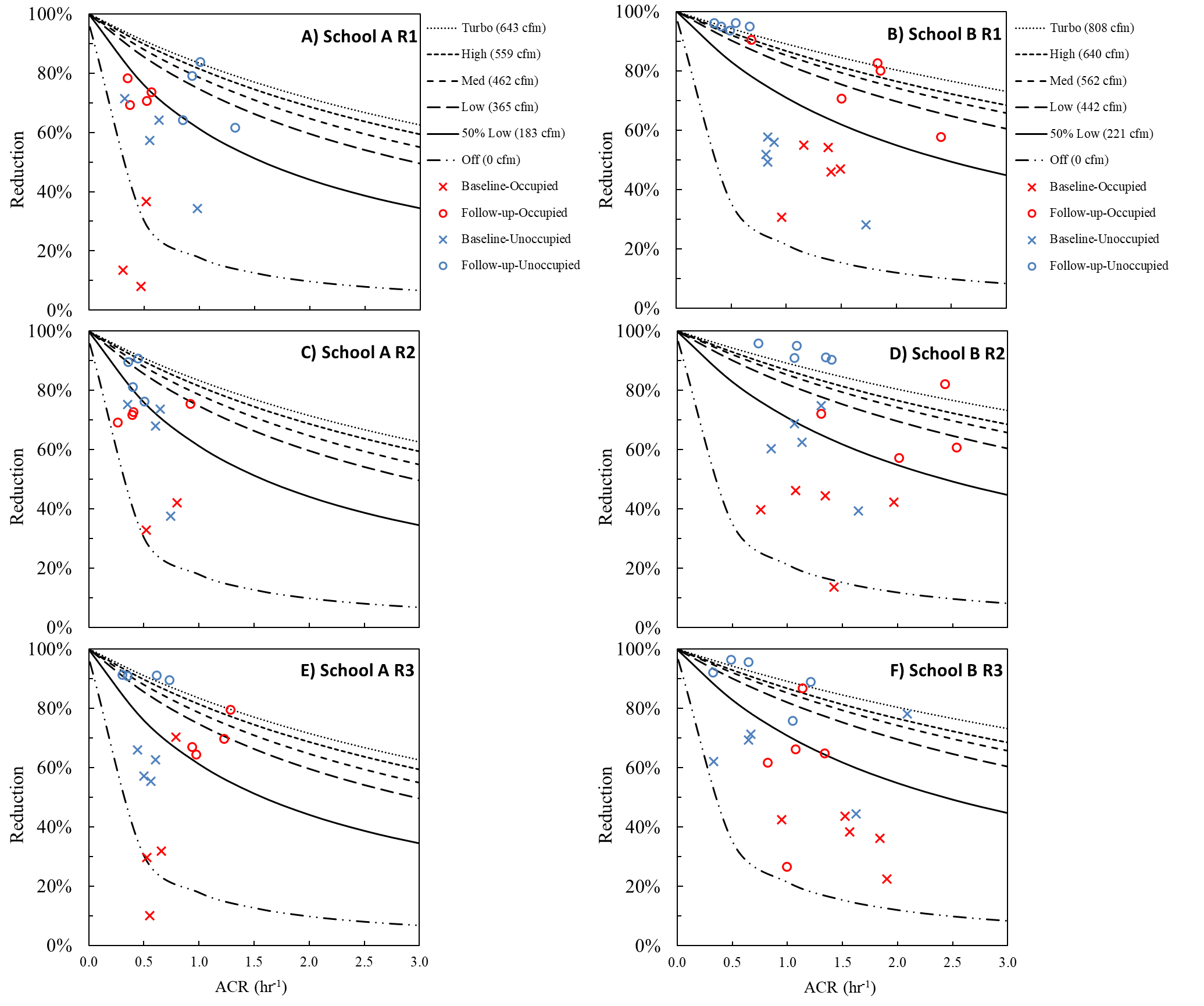


Figure S4. In-classroom PM_2.5_ reductions as a function of air change rate (ACR) and filter usage in the six examined classrooms. 1 m/s friction velocity was assumed, and the mixing factor equals 1. Crosses and circles represent daily averaged measurement results. Lines represent modeled reductions with six filter usage scenarios, assuming two Whispure units were used at the same speed in each room. 50% Low indicates the scenario with only one unit running at the low-speed setting.

1. Mixing Factor = 1


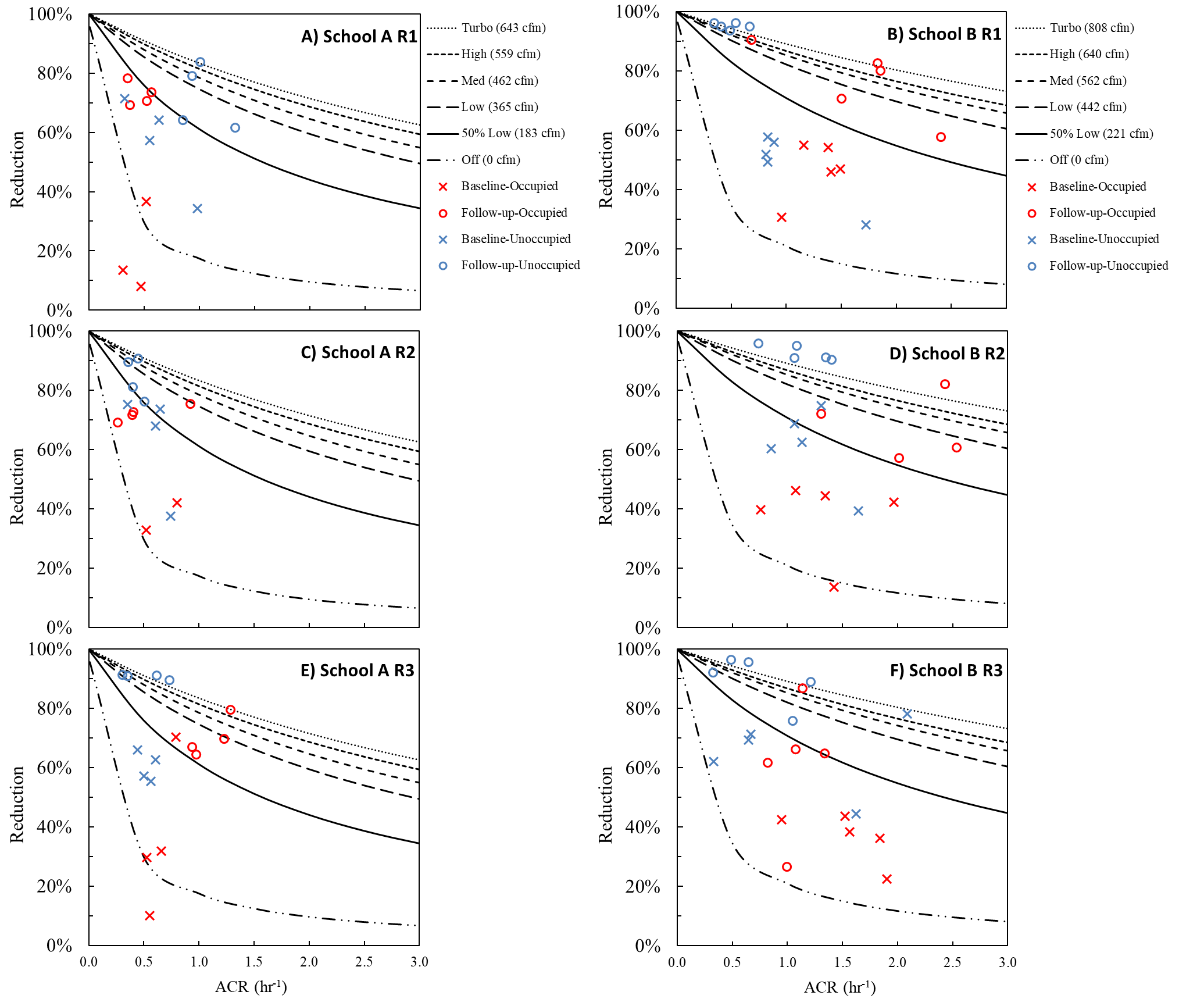


1. Mixing Factor = 0.75


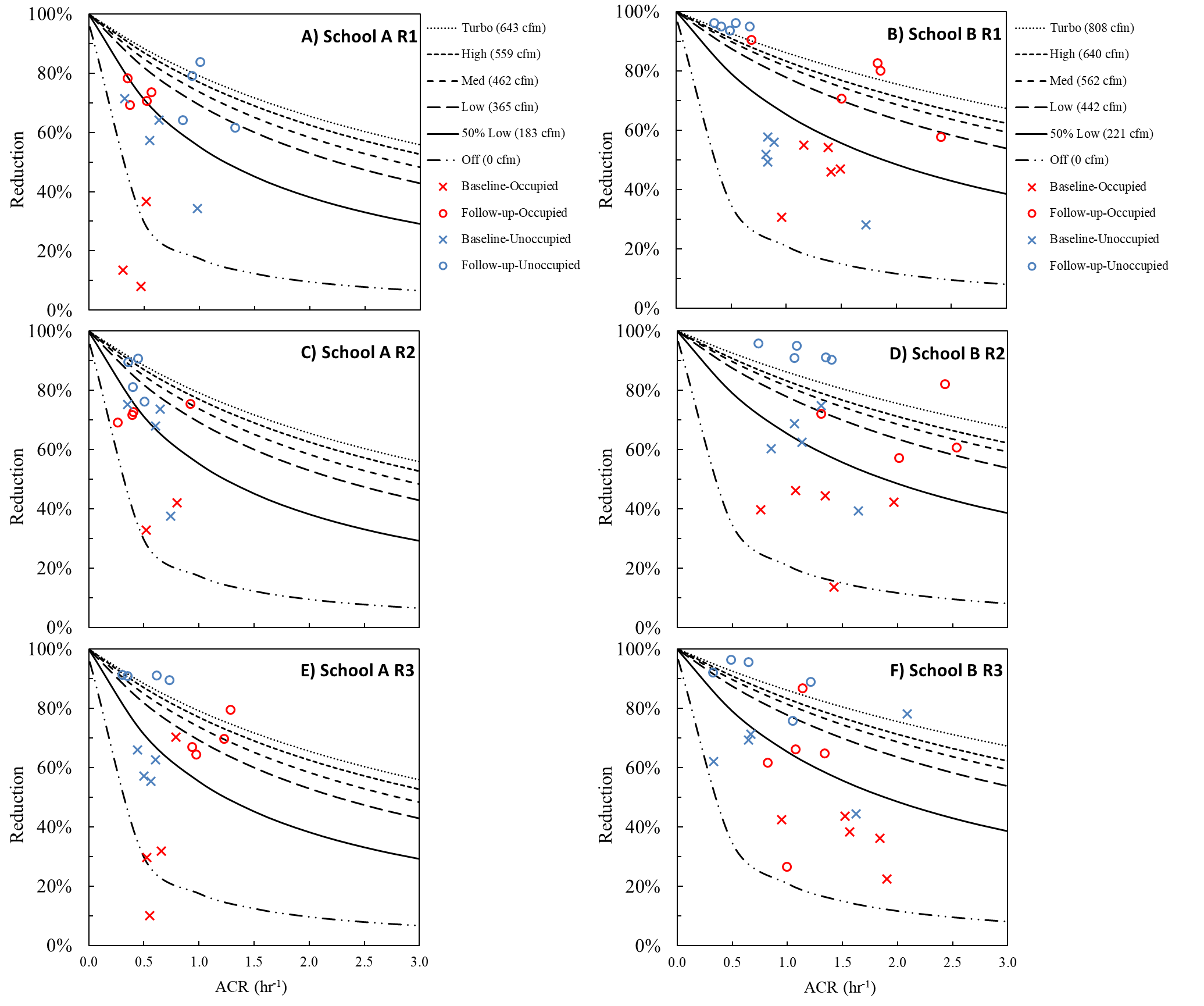


1. Mixing Factor = 0.5


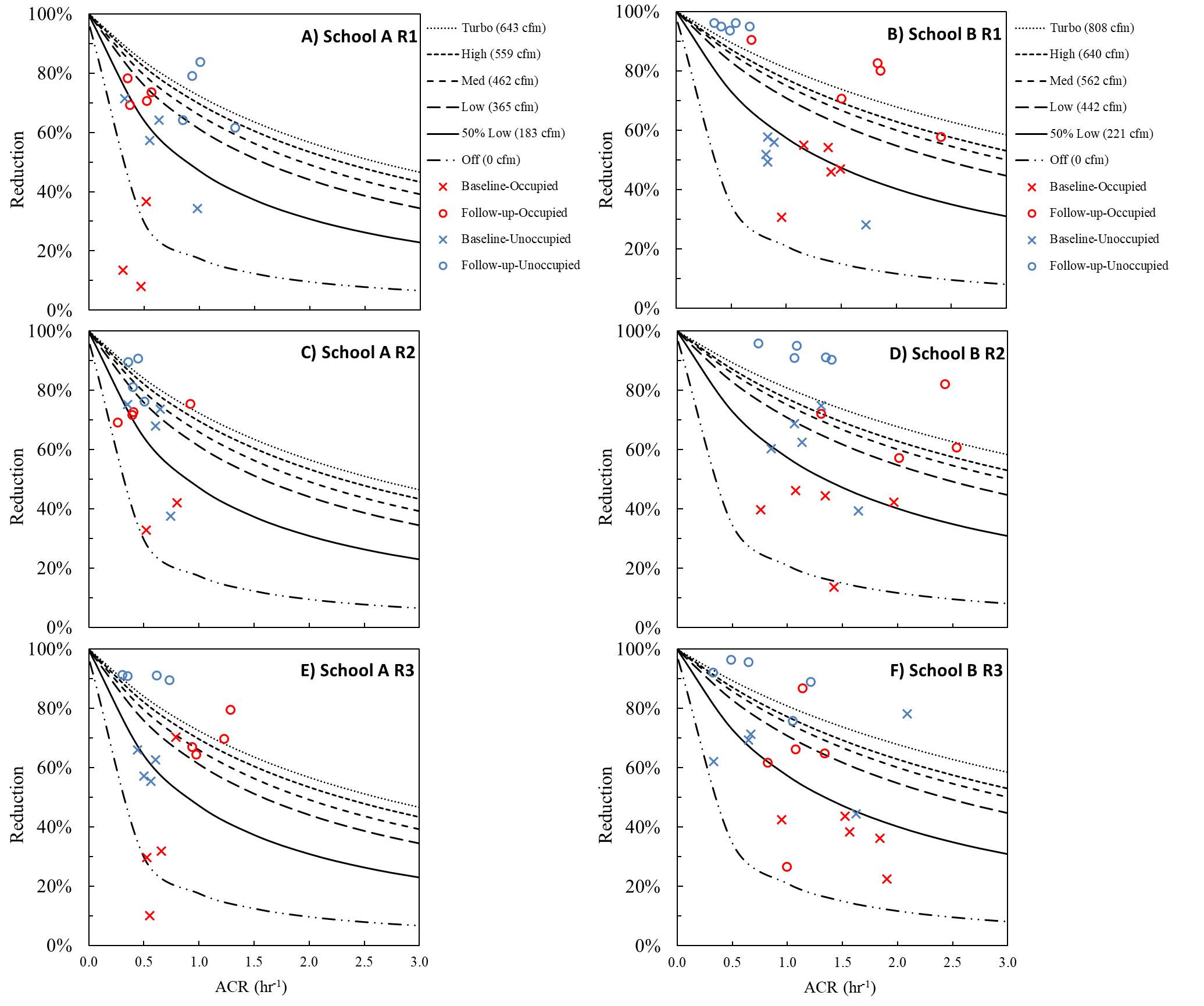


Figure S5. In-classroom PM_2.5_ reductions as a function of ACR and filter usage in the six examined classrooms with mixing factor equals a) 1, b) 0.75 and c) 0.5 for both schools. 0.3 m/s friction velocity was assumed, while the resulted curves in a) are not obviously different from Figure S2. Crosses and circles represent daily averaged measurement results. Lines represent modeled reductions with six filter usage scenarios, assuming two Whispure units were used at the same speed in each room. 50% Low indicates the scenario with only one unit running at the low-speed setting.

Mixing Factor = 1


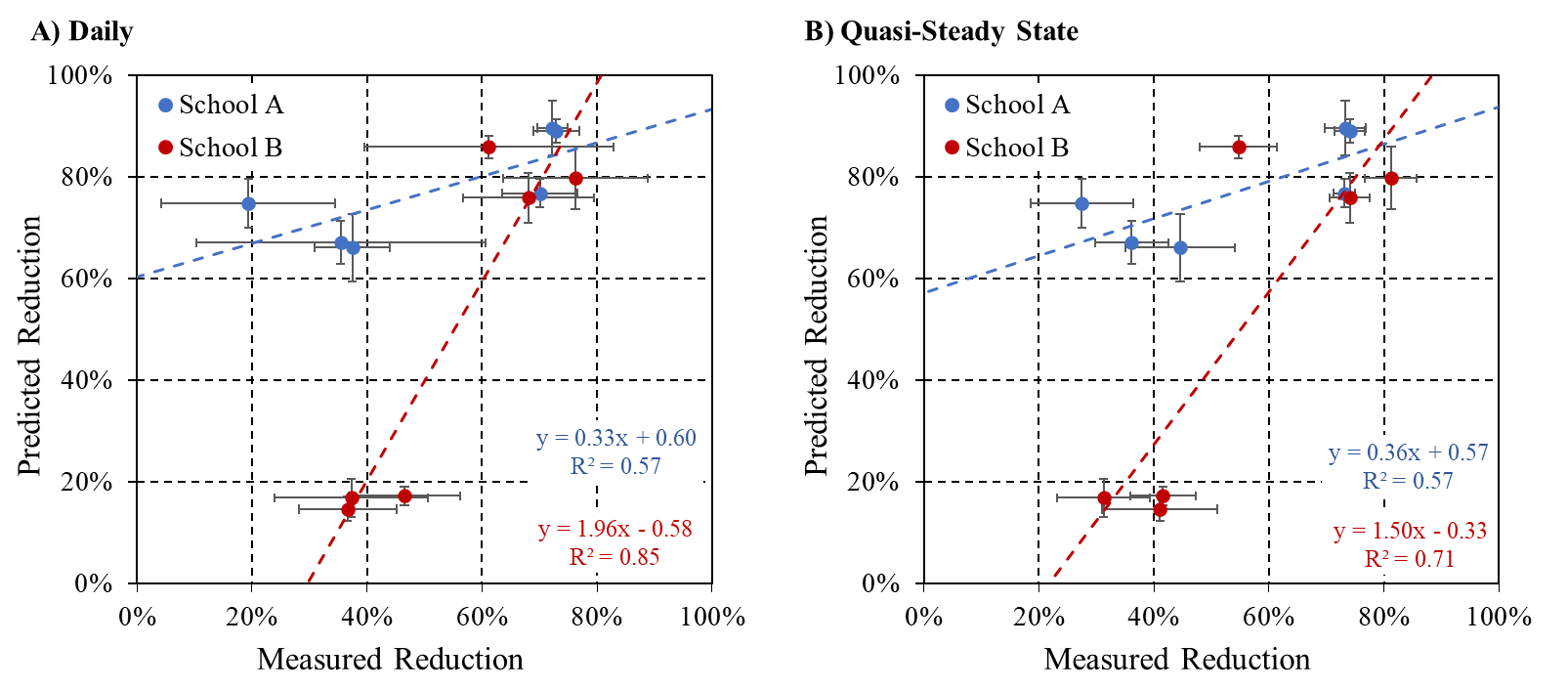


Mixing Factor = 0.75


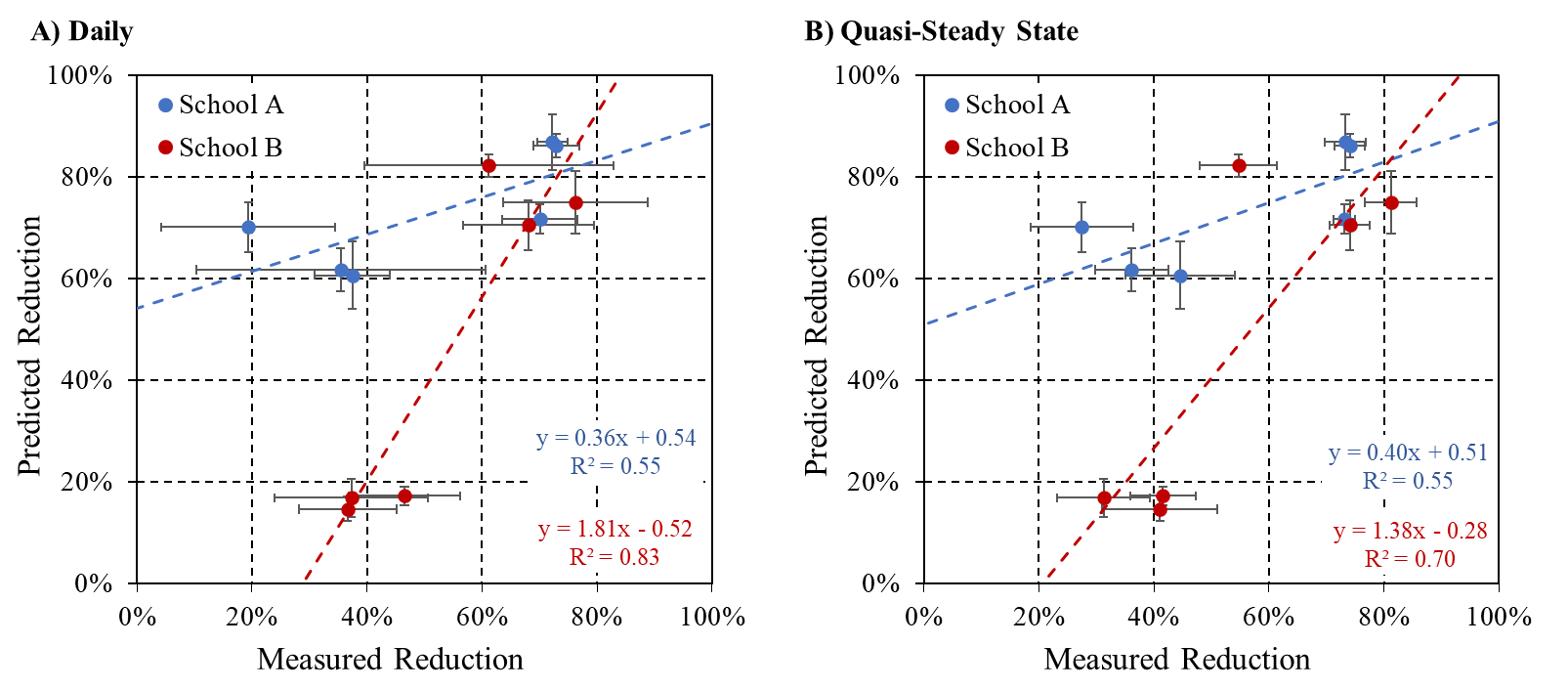


Mixing Factor = 0.5


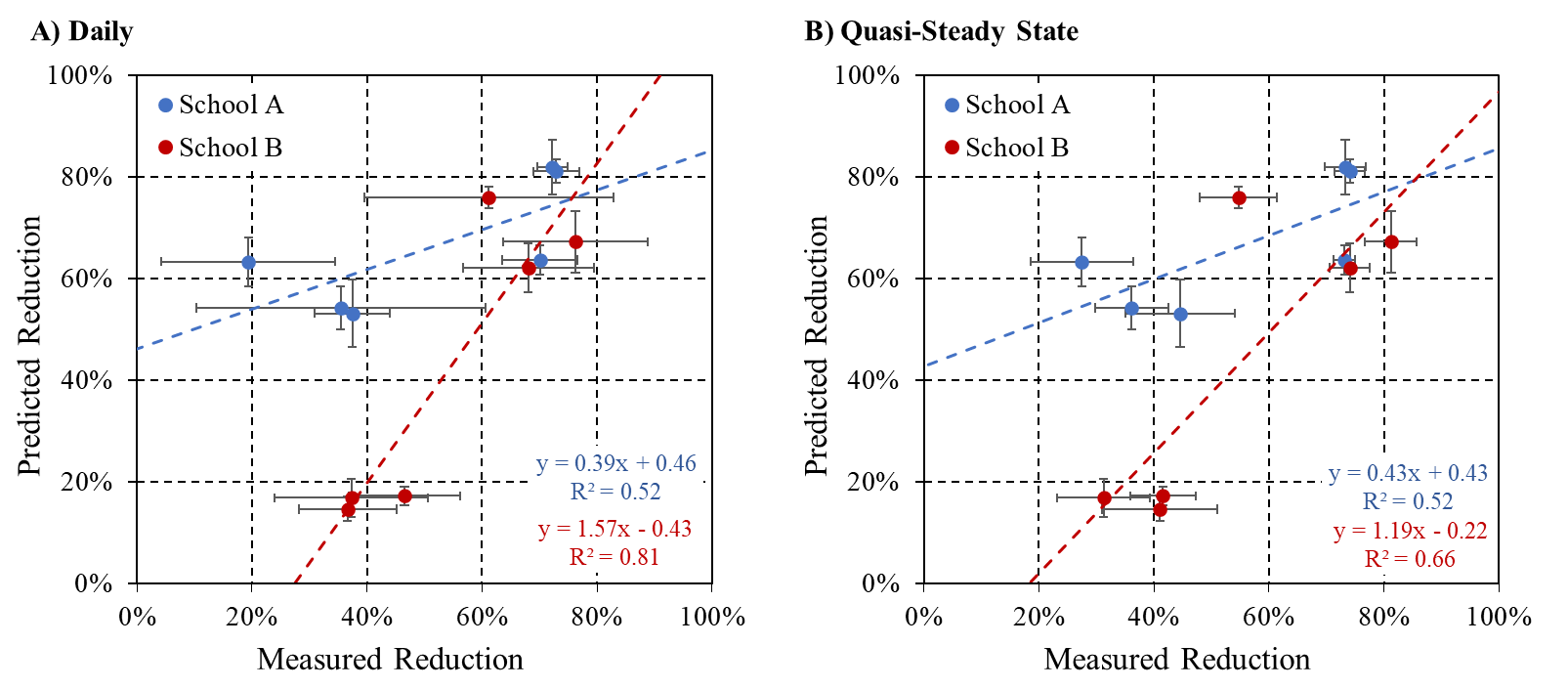


Figure S6. Average predicted PM_2.5_ removal versus the average measured removal at the six classrooms during occupied hours with three mixing factor setups using a) I/O ratio of daily median concentration and b) averaged I/O ratio during identified quasi-steady state periods. Baseline and follow-up measurements were plotted together. Linear correlations and the trendline are color-coded. The two methods yielded similar results. At school B the correlation is stronger with daily values.

# References

1. Goyal R, Khare M. Indoor air quality modeling for PM10, PM2. 5, and PM1. 0 in naturally ventilated classrooms of an urban Indian school building. Environ Monit Assess. 2011;176(1):501–16.

2. Hoang A, Heming A. Preparation in the pandemic: How schools implemented air quality measures to protect occupants from COVID-19. World Health Organ Geneva Switz. 2021;

3. Ng L, Poppendieck D, Polidoro B, Dols WS, Emmerich S, Persily AK. Single-Zone Simulations Using FaTIMA for Reducing Aerosol Exposure in Educational Spaces. NIST [Internet]. 2021 Apr 8 [cited 2024 Feb 14]; Available from: https://www.nist.gov/publications/single-zone-simulations-using-fatima-reducing-aerosol-exposure-educational-spaces

4. Lai ACK, Nazaroff WW. MODELING INDOOR PARTICLE DEPOSITION FROM TURBULENT FLOW ONTO SMOOTH SURFACES. J Aerosol Sci. 2000 Apr 1;31(4):463–76.

5. Pfeiffer T, Dilip KH, Suryawanshi R, Kishnamurthy GS, Téllez BD, Creutzburg R, et al. Airflow visualization and air purifier positioning optimization in potentially COVID-19 contaminated classrooms. Electron Imaging. 2021;33:1–15.

6. Burgmann S, Janoske U. Transmission and reduction of aerosols in classrooms using air purifier systems. Phys Fluids. 2021 Mar 23;33(3):033321.

7. Liu H. Air purifier flow field simulation and structural optimization. Int Core J Eng. 2019;5(11):66–73.

8. Liu YL, Kuan YD, Luo WJ. A Study on Flow Field Characteristics and Air Purifier with Barrier Effects. Processes. 2022 May;10(5):864.

9. Zhao W, Mustakallio P, Lestinen S, Kilpeläinen S, Jokisalo J, Kosonen R. Numerical and experimental study on the indoor climate in a classroom with mixing and displacement air distribution methods. Buildings. 2022;12(9):1314.
